# ONC201 kills breast cancer cells *in vitro* by targeting mitochondria

**DOI:** 10.18632/oncotarget.24862

**Published:** 2018-04-06

**Authors:** Yoshimi Endo Greer, Natalie Porat-Shliom, Kunio Nagashima, Christina Stuelten, Dan Crooks, Vishal N. Koparde, Samuel F. Gilbert, Celia Islam, Ashley Ubaldini, Yun Ji, Luca Gattinoni, Ferri Soheilian, Xiantao Wang, Markus Hafner, Jyoti Shetty, Bao Tran, Parthav Jailwala, Maggie Cam, Martin Lang, Donna Voeller, William C. Reinhold, Vinodh Rajapakse, Yves Pommier, Roberto Weigert, W. Marston Linehan, Stanley Lipkowitz

**Affiliations:** ^1^ Women's Malignancies Branch, Center for Cancer Research (CCR), National Cancer Institute (NCI), National Institutes of Health (NIH), Bethesda, MD, USA; ^2^ Laboratory of Cellular and Molecular Biology, CCR, NCI, NIH, Bethesda, MD, USA; ^3^ Electron Microscope Laboratory, Leidos Biomedical Research, Inc. Frederick National Laboratory for Cancer Research (FNLCR), Frederick, MD, USA; ^4^ Urologic Oncology Branch, CCR, NCI, NIH, Bethesda, MD, USA; ^5^ CCR Collaborative Bioinformatics Resource, Leidos Biomedical Research, Inc., FNLCR, Frederick, MD, USA; ^6^ Experimental Transplantation and Immunology Branch, CCR, NCI, NIH, Bethesda, MD, USA; ^7^ RNA Molecular Biology Group, Laboratory of Muscle Stem Cells and Gene Regulation, National Institute of Arthritis and Musculoskeletal and Skin Diseases, NIH, Bethesda, MD, USA; ^8^ CCR Sequencing Facility, Leidos Biomedical Research, Inc., FNLCR, Frederick, MD, USA; ^9^ Developmental Therapeutics Branch, CCR, NCI, NIH, Bethesda, MD, USA

**Keywords:** ONC201, breast cancer, mitochondria

## Abstract

We report a novel mechanism of action of ONC201 as a mitochondria-targeting drug in cancer cells. ONC201 was originally identified as a small molecule that induces transcription of TNF-related apoptosis-inducing ligand (TRAIL) and subsequently kills cancer cells by activating TRAIL death receptors. In this study, we examined ONC201 toxicity on multiple human breast and endometrial cancer cell lines. ONC201 attenuated cell viability in all cancer cell lines tested. Unexpectedly, ONC201 toxicity was not dependent on either TRAIL receptors nor caspases. Time-lapse live cell imaging revealed that ONC201 induces cell membrane ballooning followed by rupture, distinct from the morphology of cells undergoing apoptosis. Further investigation found that ONC201 induces phosphorylation of AMP-dependent kinase and ATP loss. Cytotoxicity and ATP depletion were significantly enhanced in the absence of glucose, suggesting that ONC201 targets mitochondrial respiration. Further analysis indicated that ONC201 indirectly inhibits mitochondrial respiration. Confocal and electron microscopic analysis demonstrated that ONC201 triggers mitochondrial structural damage and functional impairment. Moreover, ONC201 decreased mitochondrial DNA (mtDNA). RNAseq analysis revealed that ONC201 suppresses expression of multiple mtDNA-encoded genes and nuclear-encoded mitochondrial genes involved in oxidative phosphorylation and other mitochondrial functions. Importantly, fumarate hydratase deficient cancer cells and multiple cancer cell lines with reduced amounts of mtDNA were resistant to ONC201. These results indicate that cells not dependent on mitochondrial respiration are ONC201-resistant. Our data demonstrate that ONC201 kills cancer cells by disrupting mitochondrial function and further suggests that cancer cells that are dependent on glycolysis will be resistant to ONC201.

## INTRODUCTION

TRAIL, a member of the TNF family of ligands, causes apoptosis through activation of its receptors, death receptor (DR) 4 and DR5 [1–3]. TRAIL has been shown to induce apoptosis in a variety of transformed cells, but not most normal cells, and therefore has been studied for anti-tumor activity in clinical trials [4]. Despite its robust anti-tumor activity *in vitro* and in animal models, TRAIL ligands and DR agonistic antibodies have shown limited efficacy in clinical trials [5–7].

ONC201 (TRAIL-inducing compound 10 [TIC10], also known as NSC350625) was originally identified by a screen to find a small molecule that induces *TRAIL* expression in tumor cells, and thereby activates DRs via an autocrine or paracrine mechanism [8]. It was reported that ONC201 induces dual inhibition of Akt and ERK, resulting in dephosphorylation of Foxo3a. This resulted in translocation of Foxo3a from the cytoplasm into nucleus, where it binds to the *TRAIL* promoter to upregulate its gene transcription [8]. Currently, ONC201 is being investigated as a novel anti-tumor therapeutic agent [9, 10]. The first phase I study has indicated that it was well tolerated and achieved micromolar plasma concentrations in advanced cancer patients [11].

Recently, two independent groups reported that ONC201 induces cell death via cell stress mechanisms, independent of *TRAIL* transcription [[12, 13], reviewed in [14]]. Ishizawa *et al.* [13] found the effect of ONC201 in acute myeloid leukemia and mantle cell lymphoma cells was not dependent on either caspase-8 activation or Foxo3a-dependent transcription of *TRAIL*. Gene expression profiling analysis revealed that ONC201 induces endoplasmic reticulum (ER) stress or integrated stress response (ISR)-related genes, such as Activating Transcription Factor 4 (*ATF4*) and C/EBP-homologous protein (*CHOP*). ER stress and the ISR are caused by pathological disturbances that promote accumulation of unfolded/misfolded proteins [15–18]. To overcome these perturbations and restore cellular homeostasis, ER stress and/or the ISR activate an unfolded protein response (UPR). However, when protein misfolding is excessive, the ER stress and ISR pathways trigger cell death [15, 19, 20]. Ishizawa et al. found that ONC201 induces apoptosis by, at least in part, by ATF4 and proposed this mechanism as an atypical ISR [13]. In a parallel study, Kline *et al.* [12] investigated early events (18 and/or 48h post treatment with ONC201) that precede the inactivation of Akt and ERK, and subsequent up-regulation of *TRAIL* expression in a variety of solid tumor cancer cell lines. Like Ishizawa *et al.*, they found *ATF4, CHOP,* and a subset of genes that possess binding sites for ATF4 and CHOP were upregulated by ONC201. They showed both *ATF4* and *CHOP* play critical roles in ONC201's mechanism of cytotoxicity in these solid tumors. Thus, both studies documented a TRAIL-independent cytotoxic effect of ONC201 in cancers. However, a detailed mechanism explaining how ONC201 kills cancer cells by inducing stress proteins has yet to be established.

In this study, we tested the activity of ONC201 in multiple breast cancer and endometrial cancer cell lines. ONC201 was toxic to all cancer cell lines tested, and we found that its cytotoxicity is independent of DR4/5 and caspase activation. We found that ONC201 depleted cellular ATP. Cytotoxicity and ATP depletion were both enhanced in non-glucose medium, suggesting that ONC201 targets mitochondrial respiration. Subsequently, we observed that ONC201 decreases mitochondrial respiration, induces mitochondrial structural damage and functional impairment, and reduces mitochondrial DNA content. Furthermore, we found that cells that are not dependent on mitochondrial respiration are ONC201-resistant. Thus, our work identifies a novel mechanism of ONC201 cytotoxicity that is based on the disruption of mitochondrial function, leading to ATP depletion and cell death in cancer cells that are dependent on mitochondrial respiration.

## RESULTS

### ONC201 induces cell death in multiple breast cancer cells in a caspase-and DR4/5-independent manner

**Figure 1:**
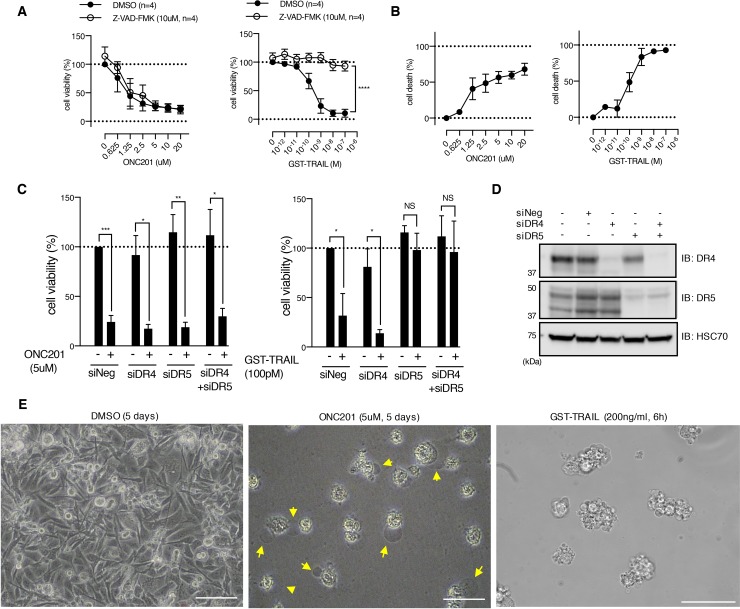
ONC201 kills breast cancer cells via caspase- and death receptor-independent mechanism **(A)** Dose-dependent effect of ONC201 and GST-TRAIL on MB231 cell viability was examined with MTS assay for 5 days treatment, in the presence or absence of Z-VAD-FMK (10μM). Results are shown as mean +/− SEM of 4 independent experiments with triplicates at each dose point. ^****^p<0.0001, two-way ANOVA. **(B)** CytoTox-Glo™ cytotoxicity assay with MB231 cells treated with either ONC201 or GST-TRAIL for 5 days. Results are shown as mean +/− SEM of 3 independent experiments with triplicates at each dose point. **(C)** Cell viability assays of MB231 cells transfected with siRNA as indicated below the graphs. Forty-eight hours after transfection, cells were treated with ONC201 or GST-TRAIL for 5 days. ^*^p<0.05, ^**^p<0.01, ^***^p<0.001, NS= not significant. Student's *t*-test. **(D)** Western blot showing siRNA-induced knockdown of DR4 and DR5. Cell lysates were collected at the same time of cell viability assay shown in C). **(E)** Light microscopy images of MB231 cells treated with DMSO (Ctl.), ONC201, or GST-TRAIL. Note that ONC201 induced cell membrane ballooning phenotype (arrows), followed by rupture (see Supplementary Movie 1-3), distinct from GST-TRAIL-induced apoptotic phenotype. Scale bar = 50μm.

We tested the effect of a 5 day exposure to ONC201 on the viability of the MDA-MB231 (MB231) triple negative breast cancer (TNBC) cell line using the MTS assay (Figure 1A, left panel). ONC201 treatment resulted in a dose-dependent decrease in cell viability with an IC50 of ~2 μM in this cell line. To ensure that the inhibition measured in the MTS assay was due to cell death, we performed a CytoTox Glo assay which measures a protease released from the cell membrane of dead cells. Again, ONC201 induced cell death with a similar IC50 (Figure 1B, left panel). We next examined the effect of ONC201 on multiple breast cancer and endometrial cancer cell lines in the MTS assay. ONC201 reduced the viability of all the breast and endometrial cancer cell lines tested. The IC50 ranged from 0.8-5 μM in breast cancer cells and 2.4-14 μM in endometrial cancer cell lines (Supplementary Table 1). All subtypes of breast cancer cells (ER+, HER2+ and TNBC) were sensitive to ONC201-mediated inhibition. Also, ONC201 inhibited viability in both serous and endometrioid subtypes of endometrial cancer (Supplementary Table 1). The IC50 seen in breast cancer and endometrial cancer cell lines is similar to that seen with the colon cancer cell line HCT116 (Supplementary Table 1), which was used in the original work describing ONC201 cytotoxicity [8]. In contrast, ONC201 did not inhibit the viability of non-transformed human foreskin fibroblast cells at concentrations up to 20 μM (HFF) (Supplementary Table 1).

ONC201 was originally reported to transcriptionally induce *TRAIL* and *DR5*, leading to DR activation and caspase-dependent apoptotic cell death [8]. Our RNAseq analysis in MB231 cells showed that ONC201 caused a decrease in the mRNA for *TRAIL* (*TNFSF10*), *DR4* (*TNFRSF10A*), *DR5* (*TNFRSF10A*), *decoy receptor 1* (*TNFRSF10C*) and *decoy receptor 2* (*TNFRSF10D*) at early time points (i.e., 3, 6 and 12 h) and only insignificant changes in *TRAIL* and modest increases in the mRNA for *DR4* (*TNFRSF10A*, 1.29 fold) and *DR5* (*TNFRSF10A*, 1.09 fold) at 24 h (Supplementary Figure 1A).

We further examined if longer treatment with ONC201 (1, 2 or 3 days) induces TRAIL, DR4, DR5 mRNA by qPCR (Supplementary Figure 1B). As a positive control, DDIT3/CHOP was also examined because our RNAseq data indicated that ONC201 strongly increases DDIT3/CHOP3 mRNA (see Supplementary Figure 2C). As an internal control, expression of glyceraldehyde-3-phosphate dehydrogenase (GAPDH) mRNA was used to normalize expression. As seen in the earlier time points in the RNAseq data in Supplementary Figure 1A for MB231, expression levels of TRAIL mRNA was decreased by ONC201 treatment (Supplementary Figure 1B). Similar decreases were seen in the SKBR3 cell line. In contrast some increase in TRAIL mRNA was seen in the MB468 and T47D cell lines but these results were not statistically significant due to large variations among the independent experiments (Supplementary Figure 1B). There were variable changes (some increase and some decreases) in DR4 and DR5 mRNA and these were mostly not statistically significant (Supplementary Figure 1B). DDIT3/CHOP3 was strongly induced in all cell lines and the elevations were statistically significant in three of the cell lines (MB231, MB468, and T47D; Supplementary Figure 1B). While DDIT3/CHOP mRNA was strongly induced in the SKBR3 cell line, the fold induction varied widely among independent experiments, making it not statistically significant (Supplementary Figure 1B).

In our previous work with breast cancer cells, the basal B TNBC were sensitive while the other subtypes of breast cancer were relatively resistant to TRAIL-induced apoptosis [1]. However, we found that ONC201 inhibited the viability of cell lines from all subtypes of breast cancer, distinct from our findings for TRAIL (Supplementary Table 1 and Supplementary Figure 1C). This is consistent with observations from a recent study by Ralff *et al*. [21]. To determine whether ONC201 initiated caspase-dependent death, we simultaneously treated cells with the pan-caspase inhibitor Z-VAD-FMK. ONC201 toxicity was not abrogated by Z-VAD-FMK in either the breast or endometrial cancer cell lines (Figure 1A left panel; Supplementary Figure 1C, 1D). In contrast, TRAIL-induced cell death was rescued by Z-VAD-FMK in all the breast and endometrial cancer cell lines tested except for T47D, which is TRAIL resistant (Figure 1A right panel; Supplementary Figure 1C, 1D). Moreover, ONC201 did not elicit PARP or caspase-3 cleavage in MB231 cells (Supplementary Figure 1E) or SKBR3 cells (Supplementary Figure 1F), while TRAIL did. These results indicate that ONC201 does not induce cell death via a caspase-dependent mechanism.

We next tested if siRNA-mediated knockdown of the TRAIL receptors, DR4 and DR5, would inhibit ONC201-induced cell death. As shown in Figure 1C and 1D, knockdown of DR4 or DR5 alone or together did not abolish ONC201-induced cell death, while knockdown of DR5 completely abrogated GST-TRAIL-dependent cell death in MB231 cells. Similarly, knockdown of DR4, DR5, or both did not rescue ONC201-induced cell death in SKBR3 cells (Supplementary Figure 1G, 1H). To test the possibility that induction of DR4 and DR5 by ONC201 treatment might overcome the siRNA-mediated knockdown of DR4 and DR5, we examined the expression of DR4 and DR5 in cells that were transfected with the siRNAs and treated with ONC201. ONC201 treatment did not result in re-expression of either DR4 or DR5 after siRNA treatment (Supplementary Figure 1I). These data demonstrate that ONC201-induced cell death is independent of TRAIL death receptors.

Time-lapse live cell imaging demonstrated that ONC201-treated cells exhibit distinct cell morphology compared to TRAIL-treated cells (Figure 1E and Supplementary Movies 1-3). ONC201-treated cells showed membrane ballooning followed by membrane rupture while TRAIL treated cells displayed classical features of apoptosis such as cell shrinkage and membrane blebbing (apoptotic body formation). This unique morphology further supported that ONC201-induced cell death is distinct from apoptosis. Taken together, these data indicate that ONC201-dependent cell death in these breast and endometrial cancer cells is not TRAIL- or caspase- mediated and morphologically it appears to be distinct from apoptosis.

### ONC201 induces AMPK activation, and ATF4 and CHOP expression, while depleting cellular ATP

**Figure 2:**
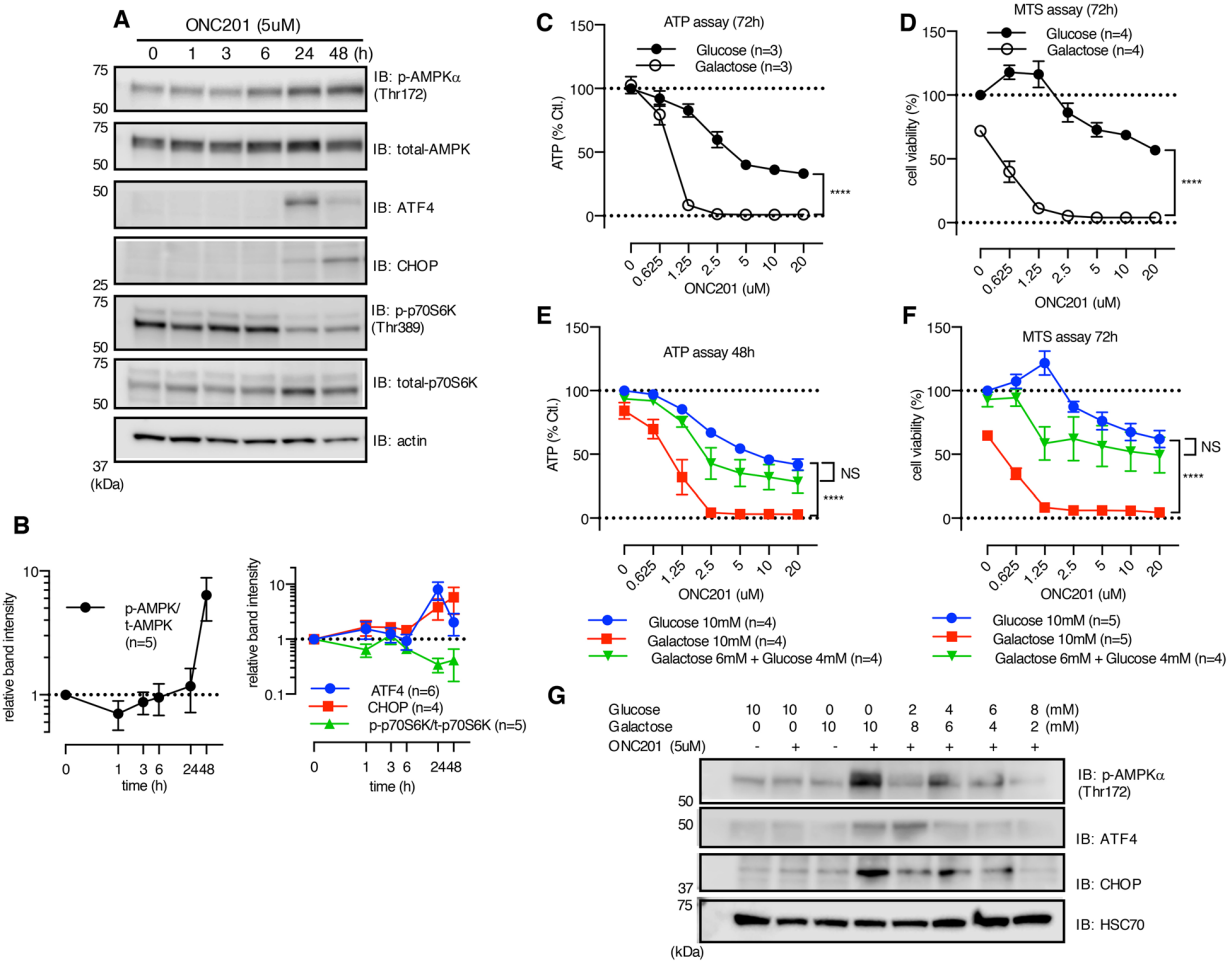
ONC201 induces AMPK activation **(A)** Western blot showing the effect of ONC201 on MB231 cells at indicated time points. **(B)** Quantitative analysis showing band intensity of p-AMPK normalized with t-AMPK (left panel), and ATF4/CHOP and p-p70S6K (right panel). ATF4 and CHOP were normalized with loading control (actin or HSC70), and p-p70S6K was normalized with total-p-70S6K. Results are shown as mean +/− SEM of multiple independent experiments. **(C** and **D)** ATP (C) and MTS assay (D) of MB231 treated with ONC201 in glucose (10mM) or galactose (10mM) medium for 3 days. Results are shown as mean +/− SEM of 3 (ATP assay) or 4 (MTS assay) independent experiments of triplicates for each dose point. ^****^p<0.0001, two-way ANOVA. **(E** and **F)** Rescue by addition of supplemental glucose of ONC201-induced reduction of ATP level (E) and cell viability (F) in galactose medium with MB231 cells. Results are shown as mean+/− SEM of 4 independent experiments. ^****^p<0.0001, NS= not significant, compared to glucose 10mM (two-way ANOVA). **(G)** Western blot showing rescue effect of supplemental glucose on ONC201-induced AMPK activation, ATF4 and CHOP induction.MB231 cells were treated with indicated conditions for 24 h. Data shown is one representative result of multiple experiments.

To investigate how ONC201 causes cell death in breast cancer cell lines, we evaluated a number of signaling pathways including those that had previously been described [8, 12, 13]. In contrast to a previous report [8], downregulation of Akt, ERK, and Foxo3a below the baseline was not observed in our model system (Supplementary Figure 2A, 2B). ONC201 did induce the stress markers ATF4 and CHOP at both the protein (Figure 2A, 2B) and message levels (Supplementary Figure 2C) and down-regulated ribosomal protein S6 kinase beta-1 (p70S6K) (Figures 2A, 2B, Supplementary Figure 2F, 2G) at 24 h or later time points, consistent with previous reports [12, 13]. Due to the non-apoptotic morphology and caspase independence of the cell death we observed above, we investigated whether ONC201 induced non-apoptotic cell death such as necroptosis or autophagocytosis. Neither necroptosis inhibitors necrostatin-1 (NEC1) nor necrosulfonamide (NSA) reversed ONC201-dependent cell death (Supplementary Figure 2D) and ONC201 did not induce changes in LC3, an autophagy marker (Supplementary Figure 2E).

In the process of examining the effects of ONC201 on a variety of signaling pathways, we found that ONC201 stimulated AMPK phosphorylation in multiple breast cancer cell lines (Figures 2A, 2B, Supplementary Figure 2F, 2G). AMPK is an “energy sensor” and phosphorylation of AMPK occurs in that response to reduction in ATP levels and other stressors [22]. Subsequent experiments confirmed that ONC201 elicited a dose-dependent decline in cellular ATP level (Figure 2C). ATP is generated by both glycolysis and mitochondrial respiration, and cells grown in glucose-containing medium can utilize both mechanisms. However, in the absence of glucose, ATP is predominantly produced by mitochondrial respiration [23–25]. ONC201-induced depletion of ATP was markedly enhanced when cells were grown in galactose-containing medium compared to glucose-containing medium in all the cell lines tested (Figure 2C, Supplementary Figure 3A). In parallel to ATP depletion, ONC201 killed MB231 cells grown in galactose-containing medium with a lower IC50 (Figure 2D). Treatment of cells with known mitochondrial targeting drugs (*e.g.*, oligomycin and metformin) also reduced cellular ATP in a dose-dependent manner, and the effect was significantly enhanced in galactose-containing medium compared with glucose-containing medium (Supplementary Figure 3A, 3B). Importantly, non-mitochondrial targeting agents, GST-TRAIL and doxorubicin, also reduced ATP levels in a dose-dependent fashion; however, this was not enhanced when cells were grown in galactose-containing medium (Supplementary Figure 3C). To examine if ONC201-induced cell death results from ATP depletion, we tested the effect of adding glucose to cells grown in galactose-containing medium. Adding glucose to galactose medium rescued ONC201-dependent ATP depletion and cell death (Figure 2E, 2F). Moreover, supplemental glucose inhibited AMPK phosphorylation, as well as ATF4 and CHOP upregulation by ONC201 when added to cells grown in galactose-containing medium (Figure 2G).

### ONC201 inhibits mitochondrial respiration

**Figure 3:**
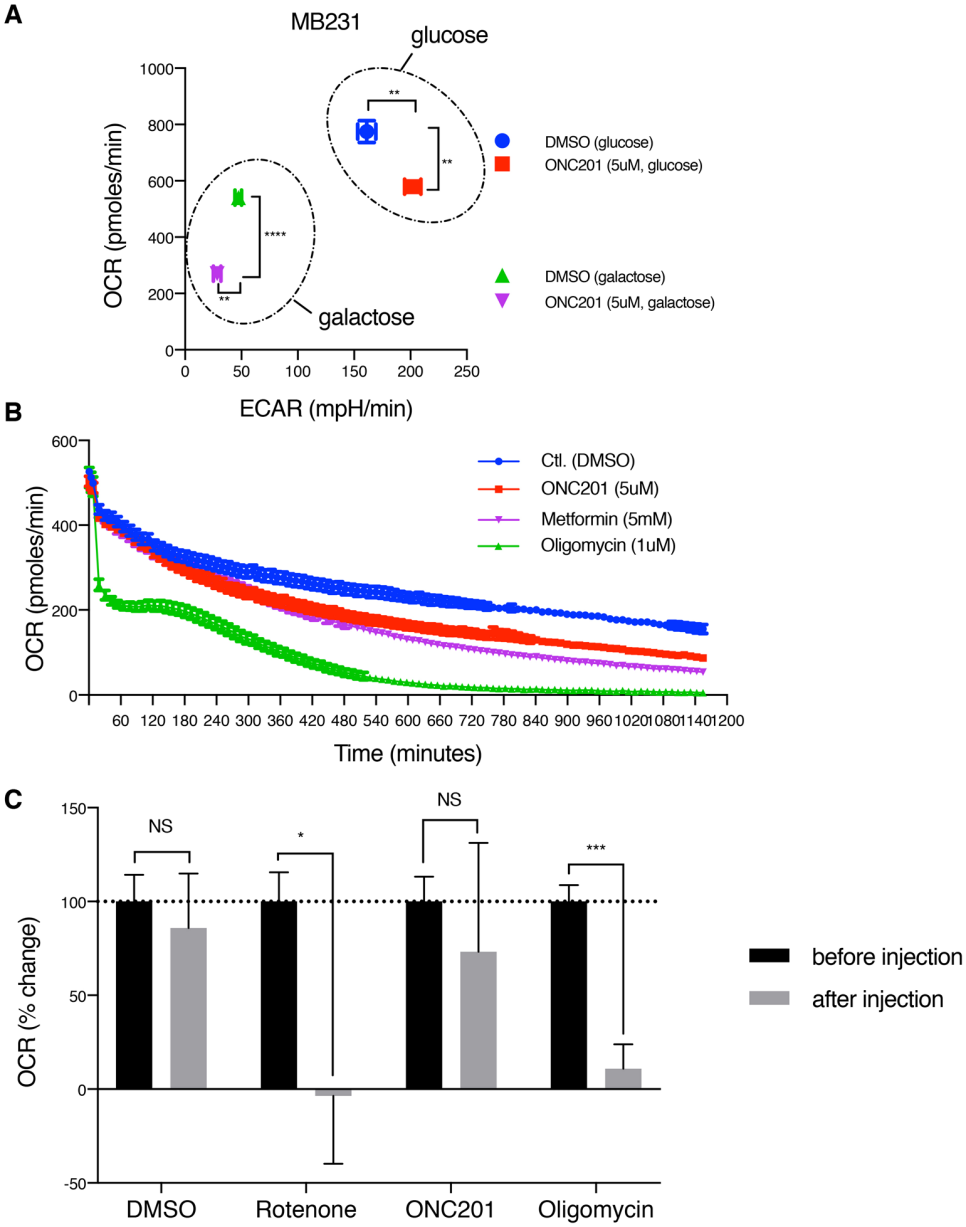
ONC201 inhibits mitochondrial respiration **(A)** OCR/ECAR measurement of MB231 cells treated with either DMSO (Ctl.) or ONC201 (5μM) in glucose or galactose medium for 24 h. OCR/ECAR were measured 5 times with 5 replicates for each condition. Data shown here is one representative result of 4 independent experiments. **(B)** Long time-course OCR measurement of MB231 cells. Drugs was injected via ports of the XF analyzer cartridge, with 5 replicates per each drug. OCR was measured every ~11 minutes and followed up to 1,200 minutes (20 h). Data is shown as mean +/− SEM. **(C)** OCR measurement with freshly isolated mitochondria from MB231 cells. Drugs were injected via ports of the XF analyzer cartridge. OCR was measured every ~3 minutes, 3 times, before and after injection (total ~20 minutes), with 4 replicates per each drug. Data is shown as mean +/− SEM of one representative result of 3 independent experiments.

The enhancement of ATP depletion by ONC201 in cells grown in galactose-containing medium suggests that ONC201 inhibits mitochondrial ATP production. Interestingly, the cytotoxic activity of ONC201 was correlated with the activity of oligomycin, a known inhibitor of oxidative phosphorylation (OxPhos) [26], when assessed on the NCI-60 panel of tumor cell lines (Supplementary Figure 3D). To further examine if ONC201 targets mitochondrial respiration, we measured the oxygen consumption rate (OCR) and extracellular acidification rate (ECAR) in cells treated with ONC201 using a Seahorse XF analyzer. In glucose-containing medium, 24 h treatment with ONC201 reduced OCR, and at the same time, increased ECAR in MB231 cells (Figure 3A). ONC201-dependent inhibition of OCR was significantly enhanced when cells were treated with the drug in galactose-containing medium, while stimulation of ECAR was not observed due to lack of glucose (Figure 3A). We next examined if ONC201 inhibits OCR acutely by adding ONC201 into permeabilized cells. ONC201 did not affect OCR or ECAR over the time course of this experiment, while two positive controls (rotenone and oligomycin, inhibitors of complex I and V, respectively) inhibited OCR within 3 minutes in MB231 cells (Supplementary Figure 3E).

Next, OCR was monitored up to 20 h after drugs were injected onto cells. For this experiment, oligomycin and metformin (a complex I inhibitor) were used as two positive controls. Oligomycin directly inhibits complex V/ATP synthase but metformin is known to slowly accumulate in the mitochondrial matrix and inhibit complex I [27, 28]. As shown in Figure 3B, oligomycin acutely decreased OCR, while both ONC201 and metformin needed 240-300 minutes to reduce OCR compared to control (DMSO), indicating that it requires time for ONC201 to inhibit mitochondrial respiration.

To further verify that the inhibitory effect of ONC201 on mitochondrial respiration is not direct, freshly isolated mitochondria from MB231 cells were used in the XF analyzer. ONC201 did not show a significant decrease of OCR, while both rotenone and oligomycin immediately inhibited OCR (Figure 3C). Together, these data suggest that ONC201 inhibits mitochondrial respiration via an indirect mechanism.

### ONC201 induces mitochondrial damage

**Figure 4:**
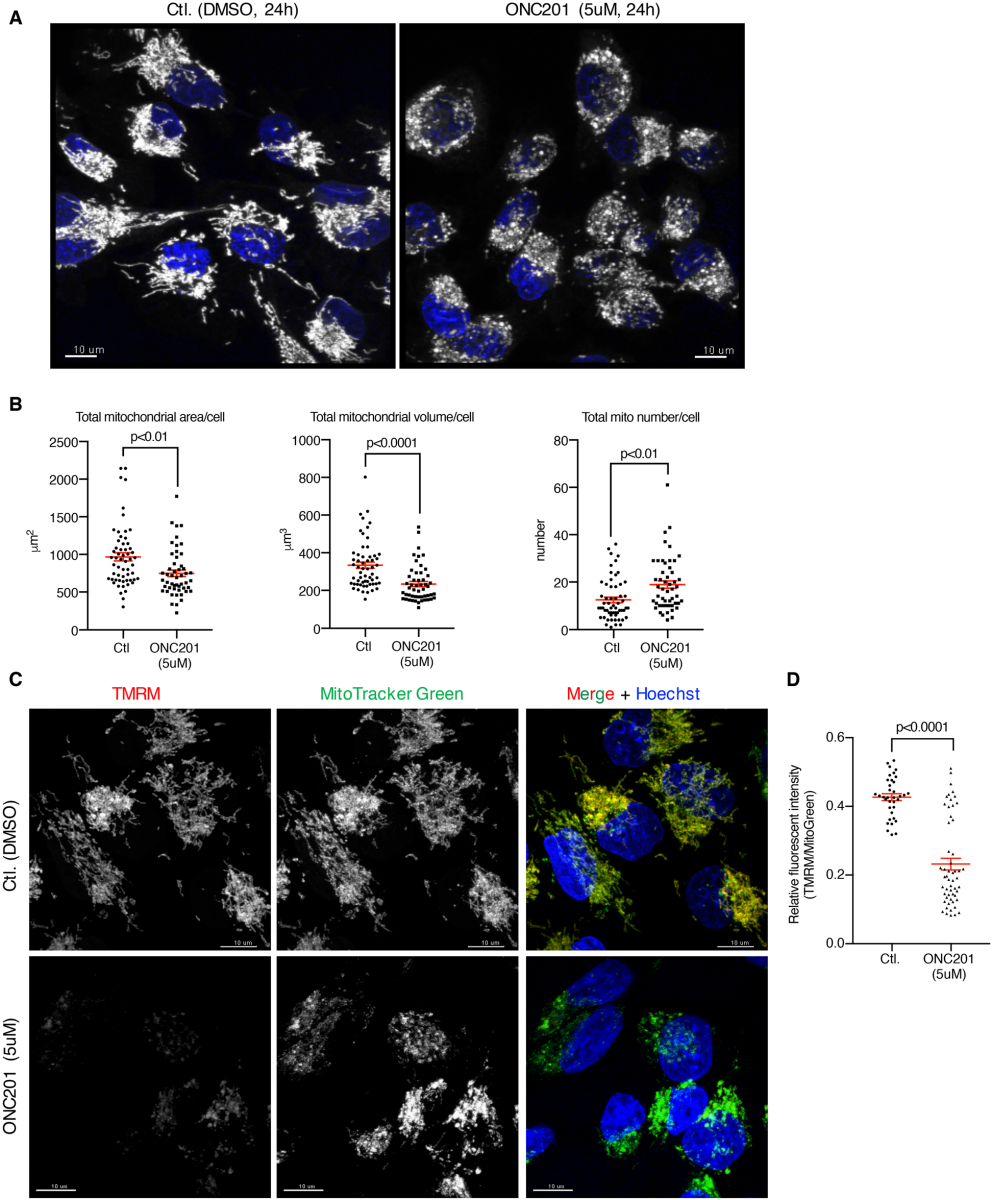

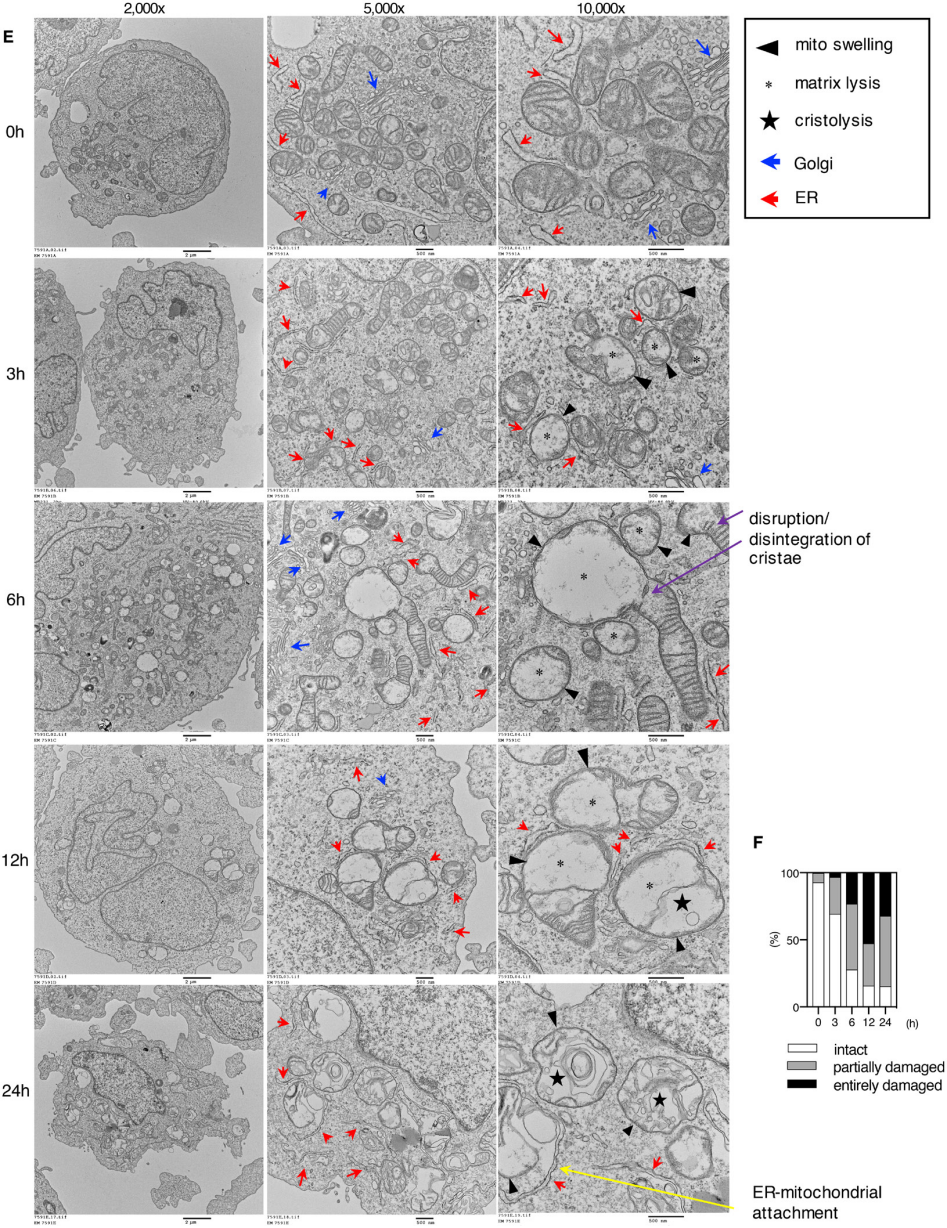
ONC201 induced mitochondrial structural damage and decreased mitochondrial membrane potential **(A)** Confocal microscopy imaging of MB231 cells treated with DMSO (Ctl.) or ONC201 (5μM). After 24 h drug treatment, mitochondria and nuclei were labeled with MitoTracker Deep Red (gray, pseudo color) and Hoechst (blue). One representative image is shown for DMSO and ONC201 each. Scale bar = 10μm. **(B)** Quantitative analysis of mitochondrial area, volume and numbers in each cell as shown in A. Total cell number examined was 58 (DMSO) and 51 (ONC201). Results are shown as mean +/− SEM. **(C)** MB231 cells were co-labeled with TMRM and MitoTracker Green for 30 minutes, then washed and treated with DMSO (Ctl.) or ONC201 (5μM) for 24 h. Cell number examined was 36 (DMSO) and 55 (ONC201). Scale bar = 10μm. **(D)** Quantitative analysis of mitochondrial membrane potential. Results are shown as mean +/− SEM. **(E)** Transmission electron microscopy images of MB231 cells treated with ONC201 (5μM) for different times. **(F)** Quantitative analysis of mitochondria visualized in (E). Two hundred mitochondria were counted at each time point. Entirely damaged, partially damaged and intact was scored if no cristae, some cristae and normal cristae were observed, respectively.

Mitochondrial morphology is tightly associated with mitochondrial health and function. Mitochondrial fusion is associated with efficient ATP production while mitochondrial fission correlates with reduced respiration and ATP production [29, 30]. To investigate the effect of ONC201 on mitochondrial shape, we performed confocal imaging analysis. Labeling of mitochondria in ONC201-treated MB231 cells revealed (24 h) mitochondrial fragmentation compared to filamentous mitochondria in control cells, (Figure 4A). In ONC201-treated cells, mitochondrial area/cell and volume/cell were both reduced, while mitochondrial number/cell was increased (Figure 4B), indicating that ONC201 induces mitochondrial fission. We tested the possibility that ONC201 promotes mitochondrial fission by targeting molecules involved with mitochondrial-fusion, such as OPA1 or Mfn2 [31]. The long forms of OPA1 (L-OPA1) and Mfn2 are known to contribute to mitochondrial fusion and their downregulation may shift the fission-fusion balance, favoring mitochondrial fission [32]. Loss of L-OPA1 and downregulation of Mfn2 were both observed at 48 h in 3 of the 4 cell lines tested (Supplementary Figure 4A). However, the mitochondrial fission shown in Figure 4A was observed at 24 h, prior to loss of these proteins. There were no clear changes in the expression of the mitochondrial fission protein, DRP1 (Supplementary Figure 4A). Moreover, mDivi-1, an inhibitor of mitochondrial fission [33], did not rescue ONC201-induced cell death (Supplementary Figure 4B). These results suggest that the mitochondrial fission-fusion machinery is not directly targeted by ONC201.

Mitochondrial membrane potential (Δψψm) is the driving force for ATP generation by mitochondria [34, 35]. We next tested if ONC201 affects Δψψm by co-labeling cells with MitoTracker Green which labels mitochondria regardless of Δψψm and TMRM that accumulates in mitochondria in a Δψψm-dependent manner. Δψψm was significantly reduced in MB231 cells after 24 h of ONC201 treatment (Figure 4C, 4D).

To further investigate the mitochondrial structural changes, transmission electron microscopy (TEM) imaging analysis was performed on MB231 cells treated with ONC201 at various time points. The TEM revealed that ONC201 induces severe mitochondrial swelling and loss of matrix (matrix lysis) as early as 3 h after addition of ONC201 (Figure 4E, 4F). At 6 h, mitochondrial swelling and matrix lysis became severe, and in longer mitochondria the damage was often focal, which appeared to result in a segmental effect (‘lollipop’ like structure). At 6 h, there was also extensive damage to cristae membranes with evidence of disruption and disintegration (see Figure 4E). In later time points, especially after 72 h, there was massive lysis of mitochondrial matrices with membrane fragments that have re-aggregated in circular forms (cristolysis; See Supplementary Figure 4C, 4D). Of note, mitochondrial damage preceded damage to the Golgi, ER, and nucleus (Figure 4E, Supplementary Figure 4C, 4D). Distention of nuclear membrane spaces was detected at 12 h (Supplementary Figure 4C). Rough ER-Mitochondrial attachment appeared to be increased, suggesting damage to the fusion proteins that maintain this interaction (Figure 4E, 24 h). Golgi complex dilatation was observed at 12 h (Supplementary Figure 4C) and extensive dilatation/vacuolation of the ER were detected at 72 h (Supplementary Figure 4D). Despite these morphological alterations, Golgi were detectable until at least until 48 h, and ER until 72 h (Supplementary Figure 4D). Some nuclei showed loss of content at 72 h (Supplementary Figure 4D, 2,000x). The ONC201-induced mitochondrial structural damage was also observed with the breast cancer cell line T47D (Supplementary Figure 4E). T47D cells showed severe mitochondrial damage/degeneration in a time-dependent manner in response to ONC201. There was a decrease in the surface area of mitochondrial cristae and mitochondrial size. Damage of mitochondria in T47D was detected as a mixed phenotype of matrix lysis, cristolysis, and fragmentation, rather than mitochondrial swelling.

### ONC201 depletes mitochondrial DNA

**Figure 5:**
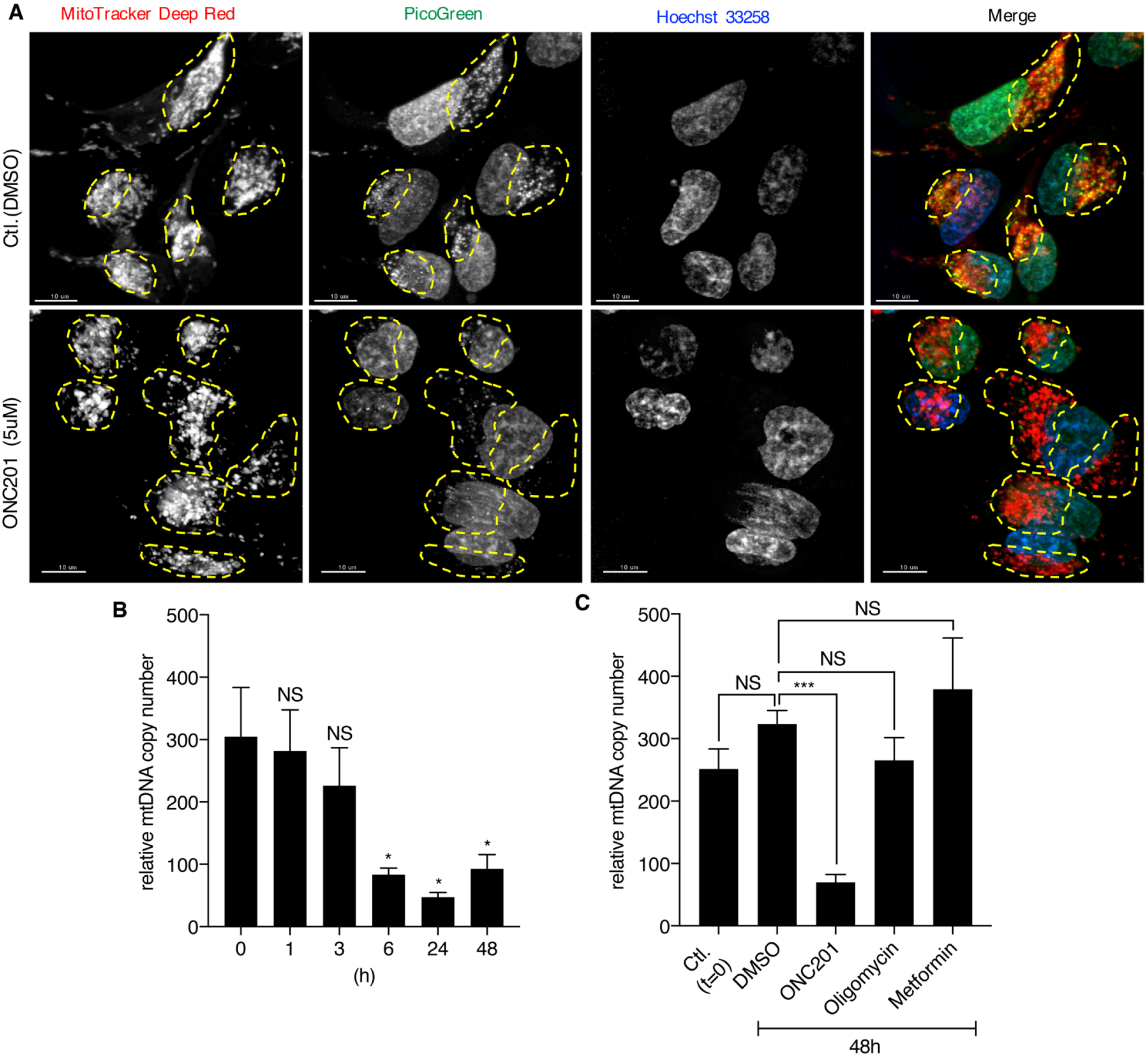
ONC201 depletes mtDNA **(A)** Confocal microscopy images of MB231 cells. Cells were treated with DMSO (Ctl.) or ONC201 (5μM) for 24 h in glucose medium, followed by co-staining with MitoTracker Deep Red (red), PicoGreen (green) and Hoechst (blue) prior to image acquisition. Mitochondria in each cell are indicated by yellow dotted circle. Scale bar=10μm. **(B)** Quantitative analysis of relative mtDNA copy number of MB231 treated with ONC201 (5μM) for different times. Results are shown as mean +/− SEM of 4 independent experiments. ^*^p<0.05, NS=not significant (*t*-test vs. time zero). **(C)** Quantitative analysis of mtDNA copy number of MB231 treated with ONC201 (5μM), oligomycin (1nM) or metformin (5mM) for 48 h in glucose medium. Results are shown as mean +/− SEM of 3 independent experiments. ^***^p<0.001, NS=not significant.

The TEM results suggested that ONC201 primarily targets mitochondria, with smaller effects subsequently observed on other organelles. Therefore, we next examined if mtDNA is affected by ONC201. Confocal microscopy imaging of cells co-labeled with MitoTracker and PicoGreen (labels both mitochondrial and nuclear DNA) revealed that ONC201 depletes mtDNA in cells treated with ONC201 for 24 h while leaving the nuclear DNA intact (Figure 5A). Quantitative PCR confirmed that ONC201 decreases relative mtDNA copy number as early as 6 h after addition of ONC201 (Figure 5B). Similar results were obtained with other breast cancer cell lines (SKBR3, T47D, BT20, Supplementary Figure 5). Notably, other mitochondrial toxins, oligomycin and metformin, did not alter relative mtDNA copy numbers (Figure 5C). This finding illustrated that ONC201 is a unique mitochondrial toxin targeting mtDNA.

### ONC201 modulates mitochondrially encoded genes and nuclear encoded mitochondrial genes

To understand gene expression alterations induced by ONC201, we performed RNAseq analysis of MB231 cells treated with ONC201 for varying times. Gene Set Enrichment Analysis (GSEA) of the RNAseq data demonstrated that ONC201 induced changes in gene expression for multiple mitochondrial processes including maintenance of the mitochondrial genome (Supplementary Figure 6A). As expected from the loss of mtDNA, many of mitochondrially encoded genes were significantly downregulated (Supplementary Figure 6B). For instance, *MT-ND6* (NADH Dehydrogenase Subunit 6), an essential component of complex I in OxPhos [36] was decreased ~20 fold at the 24 h time point. Multiple nuclear encoded mitochondrial (NEM) genes, such as mitochondrial nucleoid genes, mitochondrial ribosome genes and components of the electron transport complexes (Supplementary Figure 6C), were also significantly modulated by ONC201 after 24 h treatment. Western blotting further confirmed that ONC201 decreases OxPhos proteins, as well as endonuclease G (a NEM gene) (Supplementary Figure 7).

The mitochondrial nucleoid is a macromolecular assembly of mtDNA, proteins and mitochondrial ribosomes, and is a crucial component of the cell's homeostatic network [37]. Among nucleoid component, mitochondrial transcription factor A (Tfam), is an essential protein in maintenance of mtDNA genome integrity, copy number, mtDNA transcription and replication, and mitochondrial biogenesis [38–43]. In addition to its transcript (Supplementary Figure 6C), we observed Tfam protein was also decreased as early as 6 h after ONC201 treatment in multiple breast cancer cell lines, and it preceded that of peroxisome proliferator-activated receptor gamma coactivator-related protein 1 (PPRC) and nuclear respiratory factor 1 (NRF1), two upstream proteins vital to mitochondrial biogenesis [44–46] (Supplementary Figure 8A, 8B, 8C, 8D). This suggests that ONC201 does not inhibit the upstream signaling cascade of mitochondrial biogenesis (PPRC -> NRF1-> Tfam). Interestingly, CellMiner analysis revealed that ONC201 activity in the NCI-60 is correlated with *Tfam* expression level in multiple cancer cell lines (Supplementary Figure 8E). Our attempt to knockdown Tfam by 3 different siRNA reagents partially reproduced ONC201 effects. All 3 siRNAs decreased endogenous Tfam protein level (Supplementary Figure 8F). The Tfam siRNAs #4 and #10, but not #2, clearly induced the plasma membrane ballooning phenotype in MB231 cells (Supplementary Figure 8G). Both Tfam siRNAs #4 and #10 significantly reduced cell viability (Supplementary Figure 8H), and all three Tfam siRNAs decreased ATP levels and mtDNA copy numbers (Supplementary Figure 8I, 8J). The discordant effect of Tfam siRNA #2 compared with other two siRNAs remains unclear; however, these results suggest that knockdown of Tfam partially phenocopies the effects of ONC201 in cells.

### Primary human foreskin fibroblasts (HFF) are ONC201-resistant, despite mitochondrial damage and mtDNA depletion induced by ONC201

**Figure 6:**
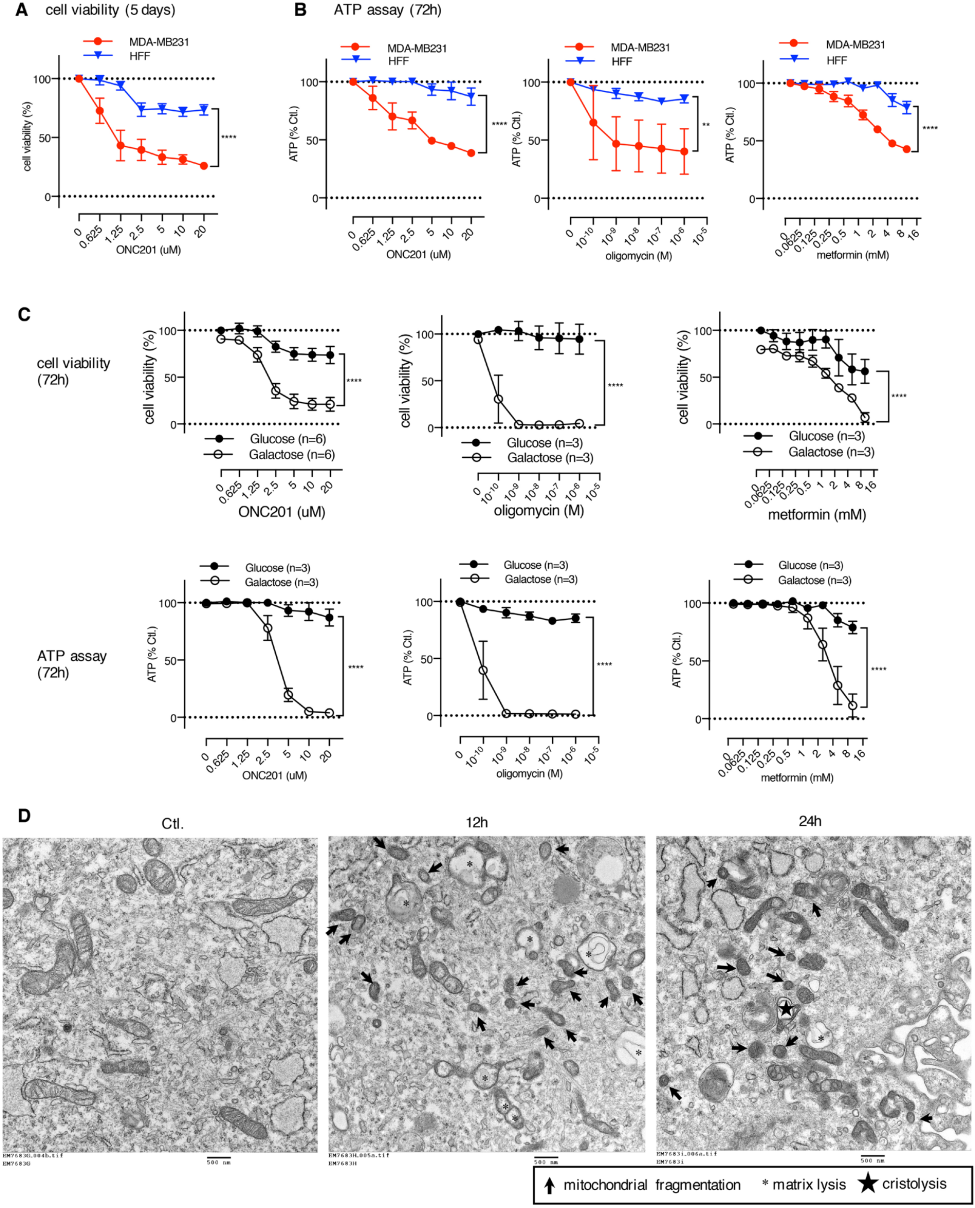

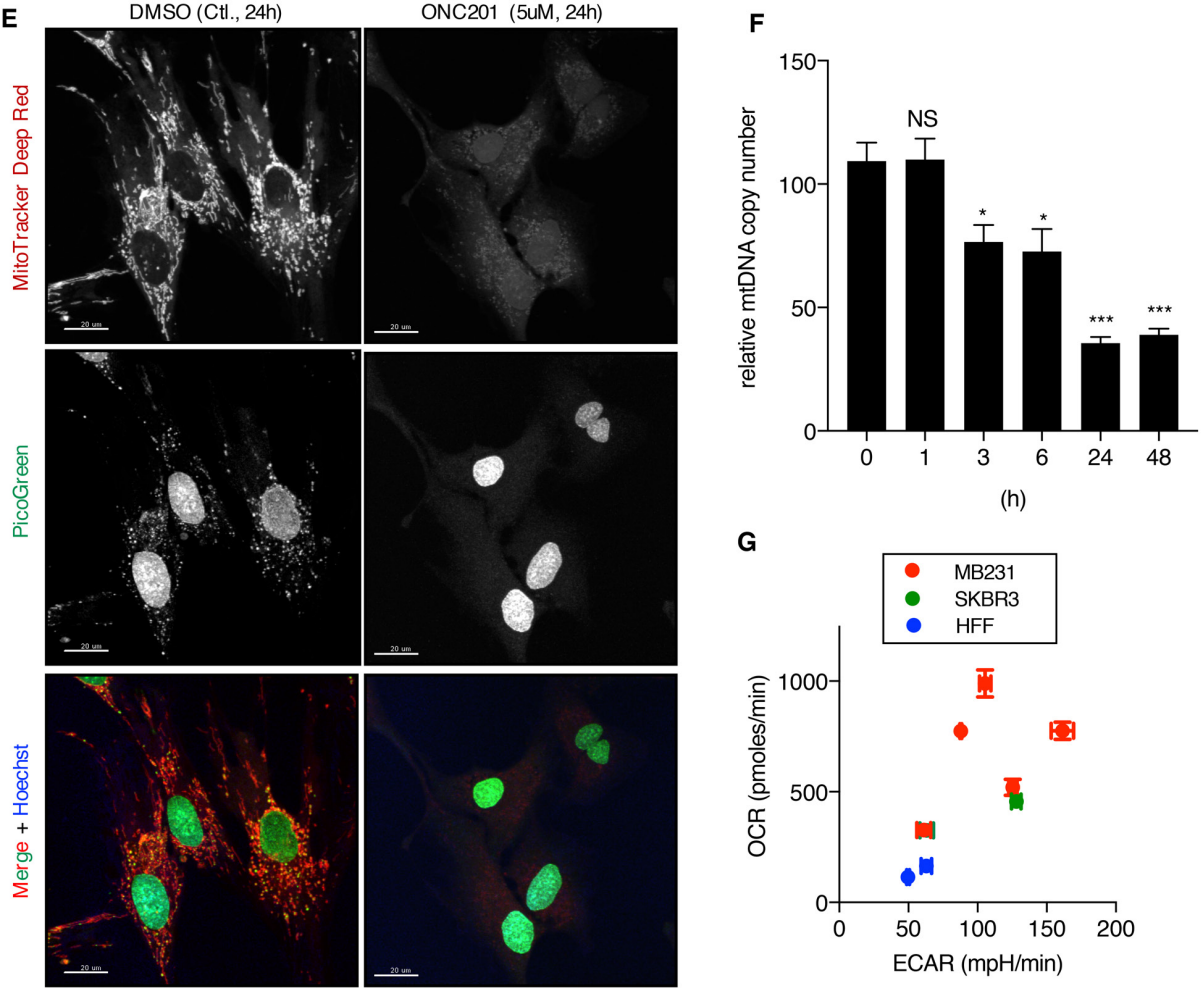
Human foreskin fibroblast cells are ONC201-resistant, despite mitochondrial damage and mtDNA depletion induced by ONC201 **(A)** Cell viability assays comparing the effect of ONC201 between MB231 and HFF cells in glucose medium. Results are shown as mean +/− SEM of 3 independent experiments. **(B)** ATP assays comparing the effect of ONC201, oligomycin, and metformin between MB231 and HFF cells in glucose medium. Results are shown as mean +/− SEM of 3 independent experiments. In both A) and B) ^**^p<0.01, ^****^p<0.0001, two-way ANOVA. **(C)** Cell viability assays (top) and ATP assays (bottom) of HFF cells treated with ONC201 and other mitochondrial targeting drugs in glucose or galactose containing medium. Results are shown as mean +/− SEM of multiple independent experiments. ^****^p<0.0001, two-way ANOVA. **(D)** TEM image of HFF cells treated with ONC201 (5μM) in glucose medium for indicated times. **(E)** Confocal microscopy image of HFF cells treated with DMSO (Ctl.) or ONC201 (5μM) for 24 h in glucose medium, and co-stained with MitoTracker Deep Red, PicoGreen and Hoechst. Scale bar = 20μm. **(F)** Quantitative analysis of mtDNA copy number in HFF cells treated with ONC201 5μM for indicated times in glucose medium. Results are shown as mean +/− SEM of 3 independent experiments. ^*^p<0.05, ^***^p<0.001 (*t*-test vs. time zero). **(G)** XF analyzer results comparing basal level of OCR/ECAR of MB231, SKBR3 and HFF cells (in glucose medium). Cell density was 100% confluent on the day of OCR/ECAR measurement. Experiments were repeated multiple times. (MB231: n=5, SKBR3: n=2, HFF: n=2).

We found thatthe non-transformed HFF cells were relatively ONC201-resistant compared to the MB231 cells in the MTS assay and an IC50 was not reached for ONC201 at the doses tested (Figure 6A). Similarly, HFF cells were resistant to ATP depletion by ONC201, as well as oligomycin and metformin compared to MB231 cells (Figure 6B). However, the HFF cells became sensitive to ONC201, oligomycin, and metformin when grown in galactose-containing medium (Figure 6C). These results imply that when grown in glucose-containing medium, the HFF cells can utilize glycolysis to produce ATP, and they become sensitive to a mitochondrial targeting drug in galactose-containing medium when the cells require mitochondrial respiration to generate ATP. TEM analysis demonstrated that ONC201 induces mitochondrial damage in HFF cells (Figure 6D). The surface area of mitochondrial cristae and mitochondrial size showed noticeable decrease. Some mitochondrial damage was detected as fragmentation and some mitochondria showed matrix lysis and cristolysis. Moreover, confocal microscopy revealed that ONC201 induces loss of mtDNA (Figure 6E, PicoGreen) and of mitochondria (Figure 6E MitoTracker Deep Red) in the HFF cells, despite the demonstrated lack of ATP depletion or cytotoxicity shown above. Quantitative PCR confirmed that ONC201 reduces the relative mtDNA copy number in HFF cells (Figure 6F). These findings indicated that ONC201 damages mitochondria and depletes mtDNA even in ONC201-resistant cells. Together, these data suggest that cells not dependent on mitochondrial respiration are ONC201-resistant. Indeed, HFF exhibited a lower OCR compared with that of MB231 and SKBR3 cells (Figure 6G), supporting the idea that HFF is not highly dependent on mitochondrial respiration.

### Cancer cells that do not depend on mitochondrial respiration are ONC201-resistant

**Figure 7:**
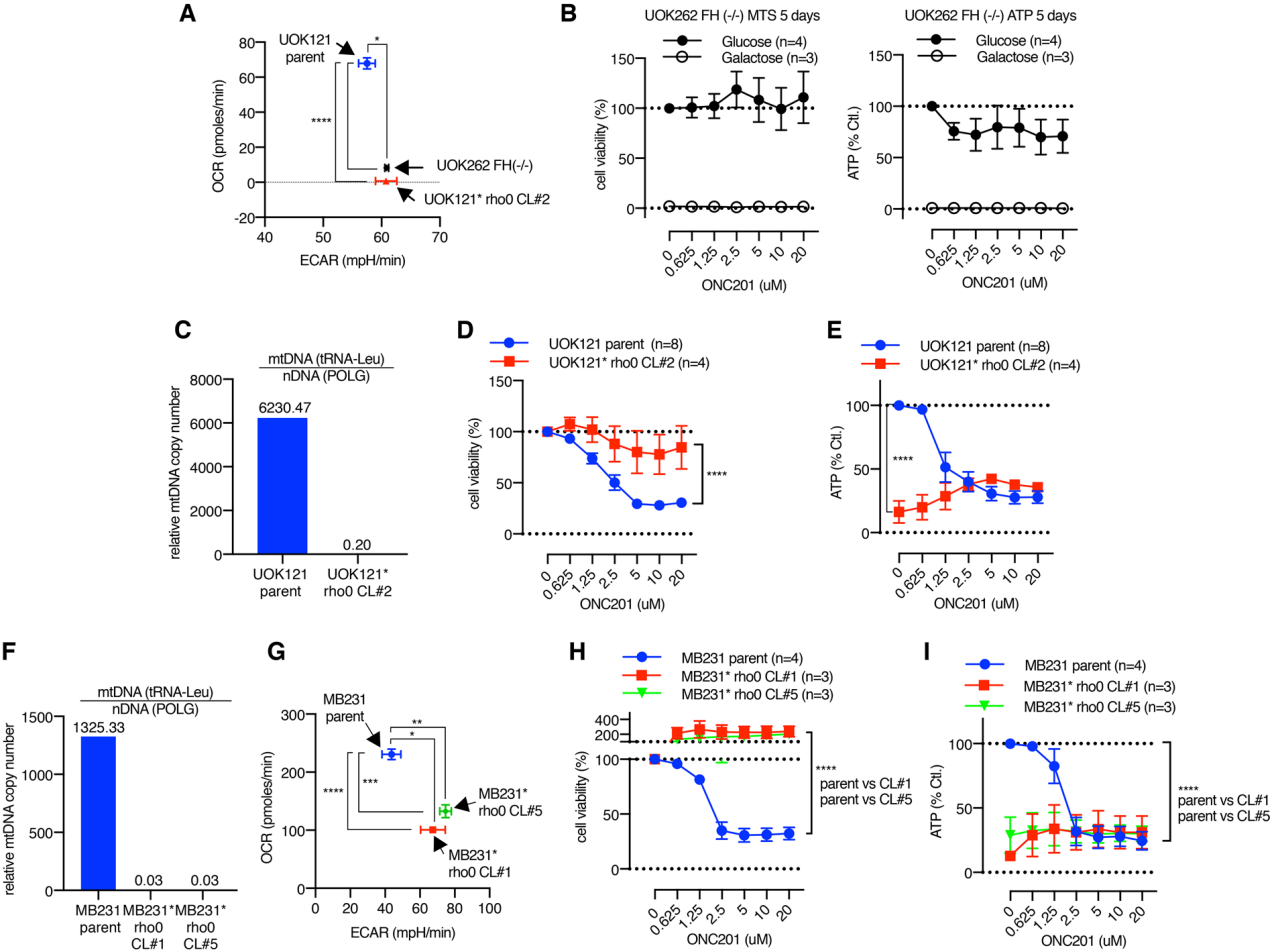
Cells with defective mitochondria are ONC201 resistant **(A)** XF analyzer result illustrating OCR/ECAR of UOK262 *FH* (−/−) cells, UOK121 parent cells, and UOK121^*^ rho0 cells. Data is shown as mean +/− SEM. ^*^p<0.05, ^****^p<0.0001, *t*-test. **(B)** Cell viability and ATP assays in UOK262 *FH* (−/−) cells. **(C)** Verification of mtDNA depletion in UOK121^*^ rho0 cells compared with UOK121 parent cells by qPCR. **(D** and **E)** The effect of ONC201 5 days treatment on cell viability (D) and ATP level (E) of UOK121 parent and UOK121^*^ rho0 cells in glucose-containing medium. Data is shown as mean +/− SEM of multiple independent experiments. ^****^p<0.0001, two-way ANOVA. Cell viability with no ONC201 was set as 100% for each cell lines in (D), ATP level of parent cells with no ONC201 was set as 100% in (E). **(F)** Verification of mtDNA depletion in MB231^*^ rho0 CL#1 and CL#5 by qPCR. **(G)** OCR/ECAR of MB231 parent and MB231^*^ rho0 CL#1 and CL#5. OCR/ECAR were measured 5 times with 5 replicates. Data shown as mean+/−SEM. ^*^p<0.05, ^**^p<0.01, ^***^p<0.001, ^****^p<0.0001, *t*-test. **(H and I)** The effect of ONC201 5 days treatment on cell viability (H) and ATP level (I) of MB231 parent and MB231^*^ rho0 CL#1 and CL#5 cell in glucose medium. Data is shown as mean +/− SEM of multiple independent experiments. Cell viability with no ONC201 was set as 100% for each cell lines in (H), ATP level of parent cells with no ONC201 was set as 100% in (I).

The above observations that HFF cells were resistant to ONC201 in glucose medium despite the mitochondrial damage raised the possibility that cells which rely on mitochondrial respiration are ONC201-sensitive, while cells not dependent on mitochondrial respiration (and dependent on glycolysis) are ONC201-resistant. To test this hypothesis in cancer cells, we examined the effects of ONC201 on UOK262 fumarate hydratase deficient (*FH* [−/−]) cells in which mitochondrial respiration is defective due to mutation of the *FH* gene. UOK262 *FH* (−/−) cells are highly glycolytic and their OxPhos is compromised [47]. Seahorse XF analysis confirmed that UOK262 *FH* (−/−) cells exhibit low OCR and relatively higher ECAR compared with UOK121 renal clear cell carcinoma cells (Figure 7A). This cell line was resistant to ONC201 reduction in viability or ATP levels when grown in glucose, and was not viable in galactose due to its dependence on glycolysis (Figure 7B).

Cells lacking mitochondria can be selected by growth in EtBr which depletes mtDNA [48]. Mitochondria deficient cells (rho0) were generated from the UOK121 renal cancer cell line (designated UOK121^*^ rho0). The UOK121^*^ rho0 cells showed lower OCR, consistent with the lack of mitochondria (Figure 7A). By contrast the parental UOK121 cell line has a higher OCR than the UOK121^*^ rho0 cells (Figure 7A). Depletion of mtDNA level in UOK121^*^ rho0 cells was confirmed by qPCR (Figure 7C). Viability and ATP levels of the UOK121 parent cells was decreased by ONC201 (Figure 7D, 7E). In contrast, the UOK121^*^ rho0 cells were ONC201-resistant in the viability assay and while they have significantly lower ATP levels than the parental cells at the basal level, ATP was not decreased by ONC201 treatment (Figure 7D, 7E). To test if depletion of mtDNA similarly makes breast cancer cells ONC201-resistant, MB231^*^ rho0 cells were generated by EtBr treatment. Quantitative PCR analysis confirmed depletion of mtDNA in two independent MB231^*^ rho0 clonal lines (Figure 7F). The two MB231^*^ rho0 clonal cell lines displayed lower OCR and higher ECAR compared to parental MB231 cells (Figure 7G). Both MTS and ATP assays confirmed that MB231^*^ rho0 cells are also ONC201-resistant (Figure 7H, 7I). As with the UOK121^*^ rho0 cells, the MB231^*^ rho0 clonal lines had lower ATP levels than the parental line, but the ATP level was not reduced by ONC201.

### Cells lacking functional mitochondria have less induction of the stress genes ATF4 and CHOP

To examine if the ONC201-induced ATF4/CHOP is due to mitochondrial stress, stress gene induction was compared between MB231 parental and MB231^*^ rho0 cells which lack functional mitochondria. ONC201 induced approximately 10 fold induction of ATF4 protein with peak induction at 24-48 h in parental MB231 cells (Supplementary Figure 10A, 10B), consistent with Figure 2A, 2B. In MB231^*^ rho0 cells, on the other hand, ONC201-induced ATF4 induction was significantly lower than parental cells (~4 fold) and induction peaked at 48h (Supplementary Figure 10A, 10B). ONC201-induced DDIT3/CHOP was analyzed by qPCR. DDIT3/CHOP was strongly increased by ONC201 in both MB231 parental cells and rho0 cells however, the induction was statistically lower in rho0 cells (Supplementary Figure 10C). Thus, the ONC201-induced induction of ATF4 and CHOP was lower in rho0 cells with non-functional mitochondria. This result suggests that functional mitochondria partly mediates the stress response, however, other non-mitochondrial mechanisms appear to also contribute to the induction of the stress response.

## DISCUSSION

In the present study, we report that ONC201 is a unique drug that targets mitochondria in cancer cells. All the breast and endometrial cancer cell lines tested were sensitive to ONC201-mediated toxicity with IC50s ranging from 0.78 to 14 μM. These are similar to the IC50s for colon, hematological, and breast cancer cells that have been previously reported [8, 13, 21, 49]. Cell lines representing all subtypes of breast cancer (ER+, HER2+, and TNBC) and serous and endometrioid endometrial cancer were sensitive to ONC201-mediated growth inhibition (Supplementary Table 1). Importantly, the IC50s observed in the breast and endometrial cells are within the range of concentrations (Cmax 3.9-19 μM) achieved in patients in a recently reported phase I study of ONC201 [11].

ONC201 was originally reported to inhibit Akt and ERK phosphorylation, leading to dephosphorylation of Foxo3a, and the transcriptional induction of *TRAIL* [8]. In MB231 breast cancer cells, we found that pAkt and pERK were slightly increased at early time points (between 1-24 h) and then decreased at later time points (48 h) and that pFoxo3a did not show significant change (Supplementary Figure 2A, 2B). Our RNAseq analysis showed that ONC201 does not induce *TRAIL* mRNA and induced only modest increases of *DR4* and *DR5* mRNA (Supplementary Figure 1A). Quantitative PCR showed decrease of TRAIL in MB231 and SKBR3 cells, and no significant changes in MB468 and T47D (Supplementary Figure 1B). Furthermore, the induction of death in the breast and endometrial cancer cell lines studies was independent of DR4, DR5, and caspase activation (Figure 1, Supplementary Figure 1). Finally, the morphological effects of ONC201 appeared distinct from TRAIL-induced apoptosis (Figure 1E and Supplementary Movie 1-3). Other investigators have found that ONC201 induces TRAIL transcription and that toxicity is in part due to TRAIL-mediated apoptosis [8, 21]. However, in the breast and endometrial cancer cells we studied, ONC201 toxicity was not mediated via the TRAIL pathway.

Our study found that ONC201 causes loss of mtDNA, morphological disruption of the mitochondria (Figures 4, 5, 6D-6F, Supplementary Figure 4C-4E, Supplementary Figure 5). The observed activation of AMPK prompted us to examine ATP depletion (Figure 2 and Supplementary Figure 3). ONC201 led to ATP depletion in parallel with loss of cell viability and this was enhanced in galactose-containing medium (Figures 2C-2F), where the primary source of ATP generation is mitochondrial respiration [25, 50]. This suggested that a primary target of ONC201 is disruption of mitochondrial ATP production. Measurement of OCR and ECAR demonstrated that the cells treated with ONC201 had decreased OCR and slightly increased ECAR (Figure 3A) in glucose-containing medium, consistent with disruption of mitochondrial respiration. The slight increase in ECAR in cells treated with ONC201 could represent a compensatory increase in glycolysis to offset the decrease in mitochondrial respiration and ATP production. Further experiments using permeabilized cells and isolated mitochondria demonstrated that the effects of ONC201 on mitochondrial respiration took time to occur and were not likely a direct inhibition of the OxPhos machinery (Figure 3C, Supplementary Figure 3E). TEM imaging demonstrated significant and rapid (3 h) morphologic disruption of mitochondria including swelling, matrix lysis and cristolysis (Figure 4E) and the morphological damage of mitochondria occurred before other organelles were affected (Figure 4E, Supplementary Figure 4C, 4D). Thus, the ONC201-induced mitochondrial damage appears to occur before the changes in stress proteins and to our knowledge represents one of the earliest effects of ONC201 described to date [8, 12, 13]. Taken together these data indicate that ONC201 disrupts the morphology and the function of the mitochondria. Consistent with the idea that ONC201 is targeting the mitochondria, ONC201 toxicity correlated with oligomycin toxicity in the NCI-60 cell line panel (Supplementary Figure 3D).

Further investigations found that ONC201 modulated expression of multiple mitochondrially encoded and nuclear encoded mitochondrial (NEM) genes at the mRNA and protein level (Supplementary Figure 6-8). We identified similar changes in mitochondrially encoded and NEM genes in the RNAseq data reported in previous papers studying colon and hematological malignancies [8, 12, 13]. This suggests that our observations are not unique to breast and endometrial cells. We also observed that ONC201 induced a rapid decrease in mtDNA after ~6 h of treatment with ONC201 (Figure 5, Supplementary Figure 5). Of particular interest was the rapid downregulation of Tfam (Supplementary Figure 8A-8D), a nuclear encoded mitochondrial transcription factor that is necessary to maintain mtDNA copy number [40]. The protein level of Tfam decreased within 6 h of treatment with ONC201 and occurred prior to the decrease in *Tfam* mRNA, suggesting ONC201 modulates Tfam via transcriptional and post-transcriptional/post-translational mechanisms. Prior studies have shown that RNAi-mediated silencing of *Tfam* results in loss of mtDNA [42, 43]. In our experiments, the loss of Tfam protein occurred in the same time frame (*e.g.*, 6h) as the decrease in mtDNA copy number (Figure 5B, Supplementary Figure 8C, 8D), similar to a previous report [42]. Silencing of *Tfam* partially phenocopied ONC201 treatment, resulting in loss of mtDNA, decrease in cellular ATP, decreased viability, and altered cell morphology similar to ONC201 treatment (Supplementary Figure 8F-8J). The activity of ONC201 and *Tfam* expression were correlated in the NCI-60 cell line panel (Supplementary Figure 8E). Together, these observations reinforce our conclusion that disruption of the mitochondria contributes to the toxicity of ONC201.

The exact mechanism leading to the changes in mitochondrial genes and protein expression induced by ONC201 is not clear. Others have proposed that ONC201 induces transcriptional changes by several mechanisms [8, 12, 13]. In the original work, the effects of ONC201 were mediated by the dephosphorylation of Foxo3a leading to translocation to the nucleus and activation of gene expression [8]. However, the changes in Foxo3a phosphorylation in the published work [8] occur 48-72 h after the addition of ONC201, while the effects on mitochondrial gene expression, morphology, and function occur earlier, suggesting that Foxo3a transcriptional activity is not directly involved in the effect of ONC201 on mitochondria. Similarly, previously reported effects of ONC201 on stress response pathways [12, 13] were induced after the mitochondrial effects that we have described. For example, we found expression of ATF4 and CHOP increased at 24-48 h in our experiments (Figures 2A, 2B, Supplementary Figure 2D). More recently, ONC201 has been proposed to function as an antagonist of dopamine receptor D2 (DRD2) [51]. *DRD2* transcripts were undetectable in the many of the breast cancer cell lines we tested (Supplementary Figure 9), suggesting that DRD2 is not likely to be the ONC201 target responsible for the effects we have observed.

**Figure 8:**
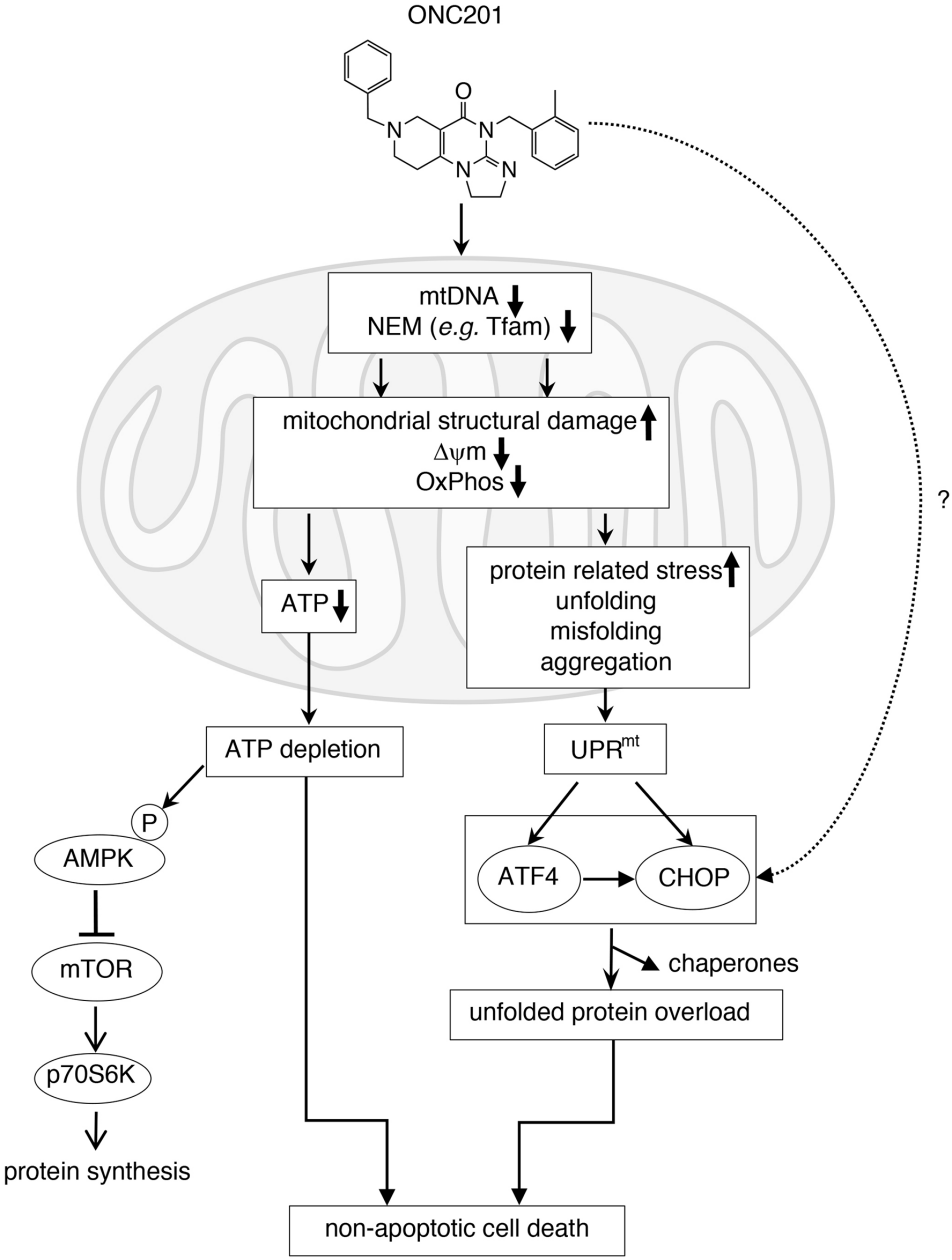
Proposed mechanism of action of ONC201 (see detail in Discussion)

Elucidating the mechanism(s) responsible for the mitochondrial damage caused by ONC201 will require further research. A proposed model for the effects of ONC201 is shown in Figure 8. ONC201 leads to transcriptional and post-transcriptional or post-translational changes in both mitochondrial and NEM genes including *Tfam*. This results in loss of mtDNA, disruption of mitochondrial structure and decreased mitochondrial membrane potential (ΔψΨm), resulting in defective mitochondrial OxPhos, and ultimately to the depletion of cellular ATP. ATP depletion promotes AMPK activation and downregulation of mTORC signaling [52, 53]. Dysfunctional mitochondria promote the accumulation of misfolded/unfolded/aggregated proteins within mitochondria, which triggers the mitochondrial unfolded protein response (UPR^mt^), leading to induction of stress proteins such as ATF4 and CHOP [54–57]. When glucose was added back to the galactose-containing medium, ATP depletion, loss of viability and stress proteins induction were rescued (Figure 2E-2G). This suggests that the activation of the stress response proteins is in part downstream of the mitochondrial disruption and ATP depletion. Stress response gene induction was significantly lower in MB231^*^ rho0 cells compared with MB231 parental cells (Supplementary Figure 10), reinforcing the hypothesis that functional mitochondria are involved in the ONC201-induced stress response. However, some induction is still present in the absence of functional mitochondria suggesting other non-mitochondrial mechanisms contribute to the induction of the stress response. ONC201-induced ATP depletion may ultimately lead to the loss of cell membrane integrity seen in the time-lapse live cell imaging due to inability of the cells to maintain osmotic gradients (Figure 2E and Supplementary Movie 2) [58].

Our investigation revealed that non-transformed HFF cells were resistant to ONC201 as well as other mitochondrial targeting drugs when grown in the presence of glucose (Figure 6). In glucose-containing medium, the HFF cells had a lower OCR compared to the ONC201-sensitive cancer cell lines (Figure 6G). This suggested that the HFF cells are not primarily dependent on OxPhos for ATP production when grown in glucose (Figure 6). Presumably, this accounts for the resistance of HFF cells to ONC201 treatment, despite the evidence that ONC201 disrupted the mitochondrial structure and depleted mtDNA in these cells (Figure 6D-6F). When the HFF cells were grown in galactose-containing medium that rendered them dependent on mitochondria for ATP production, they became sensitive to ONC201 and other mitochondria-targeting drugs (Figure 6C). These results implied that cells dependent on glycolysis for ATP production would be resistant to ONC201 cytotoxicity. To test this idea, we examined the effects of ONC201 on renal and breast cancer cells that lack mtDNA or that have a mutation in *FH*, a protein essential to the tricarboxylic acid (TCA) cycle. These cells all had relatively low OCR and high ECAR (Figure 7A, 7G), consistent with their predicted dependence on glycolysis for ATP production and were ONC201-resistant (Figure 7B, 7D, 7E, 7H, 7I).

Our results establish that ONC201 targets mitochondria, and also challenge the “Warburg effect”, a concept that cancer cells are dependent on glycolysis (reviewed in [59]). Recent studies have led to a new model for understanding the Warburg effect as it applies to tumor metabolism for breast cancer and lymphoma [60–62]. According to this revised hypothesis, epithelial cancer cells induce the Warburg effect (aerobic glycolysis) in neighboring stromal fibroblasts. The cancer-associated fibroblasts secrete lactate and pyruvate (energy metabolites resulting from aerobic glycolysis) that are taken up by epithelial cancer cells and used as fuel in the mitochondrial TCA cycle, thereby promoting efficient energy production (ATP generation via OxPhos), which enables a higher proliferative capacity. In essence, fibroblastic tumor stroma would directly feed the epithelial cancer cells, in a type of host-parasite relationship (a so-called “Reverse Warburg Effect”). This alternative model is still consistent with Warburg's original observation that tumors (which consist of cancer cells and stromal cells) show a metabolic shift towards aerobic glycolysis. This concept is consistent with our findings that breast and endometrial cancer cell lines are dependent on mitochondrial respiration. ONC201 sensitivity via mitochondria targeting might extend to other cancers. Indeed, our analysis of published gene expression data in other tumor types (colon cancer cell line HCT116 in [8] and mantle cell lymphoma Jeko-1 in [13]) found that ONC201 induced decreases in mitochondrial gene expression. Our results also predict that the sensitivity or resistance of cancer cells in patients to ONC201 may depend on the relative dependence of their tumors on OxPhos or glycolysis, respectively. Future work will explore the relevance of our findings *in vivo*. In a recent first-in-human phase I study, no serious toxicity for ONC201 has been detected in patients [11]. Careful and detailed metabolic monitoring will be required to determine if ONC201 affects mitochondrial function in cancer patients.

In conclusion, we report that ONC201 has a novel mechanism whereby it disrupts mitochondrial function and results in cell death in breast and endometrial cancer cells. While the detailed mechanism of how ONC201 impairs the mitochondria requires further study, our findings suggest that ONC201 is a promising novel anti-cancer therapy for targeting cancer cells that are dependent on mitochondria.

## MATERIALS AND METHODS

### Reagents

ONC201 was generously provided by Oncoceutics, Inc. Philadelphia, PA. ONC201 was dissolved with DMSO at 20mM and stored at −30°C. Recombinant GST-TRAIL protein was prepared in the lab as previously reported [63] and stored at −80°C in aliquots until used.The tetra-peptide pan-caspase inhibitor Z-VAD-FMK (cat#2163), necroptosis inhibitor necrostatin-1 (NEC1, cat#2324), necrosulfonamide (NSA, cat#5025), tunicamycin (cat#3516) were obtained from TOCRIS (Bristol, UK). Oligomycin (cat#O4876; stock 10mM in DMSO), metformin-hydrochloride (cat#PHR1084: stock 1M in PBS), rotenone (cat#R8875; stock 2mM in DMSO), and D-(+) galactose (cat#G5388), SP600125 (cat#S5567), ethidium bromide (EtBr, cat#E1510), Mdivi-1 (cat#M0199), and uridine (cat# U3003) were from Sigma-Aldrich (St. Louis, MO, USA). D-(+) glucose (cat#4912) was from Thomas Scientific (Swedesboro, NJ, USA). Doxorubicin (cat#S1208) was from Selleck Chemicals (Houston, TX, USA).

### Cell culture

All human breast cancer cell lines (T47D, MCF7, HCC1500, ZR75-1, BT474, SKBR3, HCC1954, AU565, HCC1937, MDA-MB468, HCC1187, BT20, MDA-MB231, Hs578T, MDA-MB436, HCC38, BT549) were grown in RPMI1640 supplemented with 10 % fetal bovine serum (FBS) and 100 units/ml of penicillin, 100 microgram/ml of streptomycin (P/S) at 37°C, 5 % CO_2_ incubator. Endometrial cancer cells ARK-1, ARK-2 were obtained from Dr. Alessandro Santin (Yale University), NCI-EC1 was from Dr. John Risinger (Michigan State University) and CRL1622 was obtained from ATCC (Manassas, VA). ARK-1 and ARK-2 (serous) were grown in RPMI1640 supplemented with 10% FBS and 1% P/S. NCI-EC1, CRL1622 (endometrioid) were grown in DMEM/F12 medium supplemented with 10% FBS and 1% P/S. Cell authenticity of each breast cancer and endometrial cell line was confirmed by Power Plex 16 (Laragen Inc., Culver City, CA, USA). Primary human foreskin fibroblast (HFF) cells [64] were kindly provided by Dr. Kenneth Yamada (National Institute of Dental and Craniofacial Research, NIH) and were maintained in DMEM supplemented with 10 % FBS and 1% P/S at 37°C, 10 % CO_2_ incubator. HCT116 was also maintained in DMEM supplemented with 10 % FBS and 1% P/S at 37°C, 10 % CO_2_ incubator. Glioblastoma stem cell line (GSC) was kindly provided by Dr. Deric Park (Neuro-Oncology Branch, NCI), and maintained in DMEM/F12 supplemented with B-27™ (final 1x, cat# 12587010, ThermoFisher), Epidermal Growth Factor (20ng/ml), Fibroblast Growth Factor (20ng/ml). UOK121 (renal cell carcinoma) and UOK262 fumarate hydratase (*FH*) deficient cells (hereditary leiomyomatosis renal cell carcinoma) [47] were maintained in rho0 medium (see *Generation of rho0 cells*section below). Cell authenticity of renal cancer cell lines were verified by Genetica Cell Line Testing, a LabCorp brand (Burlington, NC, USA).

### Cell viability assay (MTS assay)

Cells were seeded at 5,000 cells/well in a 96 well plate on the day before treatment. ONC201 or GST-TRAIL-mediated cytotoxicity was assessed using the Cell Titer 96® AQueous One Solution Cell Proliferation Assay (cat#G3581, Promega, Madison, WI, USA) as previously described [65]. All MTS measurements were done in 3 replicates and each experiment was carried out at least 3 times. Results were given as the mean +/− the standard error of the mean (SEM) for at least 3 independent experiments. When Z-VAD-FMK was used, cells were pretreated with Z-VAD-FMK (10 μM) for 30 minutes prior to any treatments. Control cells were incubated with DMSO at the same concentration as in the Z-VAD-FMK-treated cells. Cell viability was analyzed by the MTS assay after 5 days of incubation with ONC201 or GST-TRAIL.

### CytoTox-Glo^TM^ cytotoxicity assay

Cells were seeded at 5,000 cells/well in a white 96 well plate (cat#07-200-566, Fisher Scientific, Hampton, NH, USA) on the day before treatment. ONC201 or GST-TRAIL-mediated cytotoxicity was also assessed by CytoTox-Glo™ Cytotoxicity Assay (cat#G9290, Promega, Madison, WI, USA). All measurements were done in 3 replicates and each experiment was carried out at least 3 times. Results were given as the mean +/− SEM for at least 3 independent experiments. Cell viability was analyzed after 5 days of incubation with ONC201 or GST-TRAIL.

### siRNA transfection

Cells were reverse-transfected with siRNA with Lipofectamine® RNAiMax (cat#13778150, ThermoFisher Scientific, Waltham, MA, USA), as recommended by the manufacturer's protocol. Briefly, for each siRNA (22.5μl of 20μM stock), RNAiMax (22.5μl) and Opti-MEM (4450 μl) were added and incubated for 20 minutes at room temperature (RT) (Mix1 4.5ml total). Cell suspensions were prepared at 10^5^cells/ml (Mix2). Mix1 and Mix2 were then combined with equal volume (4.5 ml + 4.5 ml), incubated for 5 minutes at RT, and plated 100 μl/well in 96 well plate for cell viability assay. Volume was changed as needed with the same ratio of each component (e.g. siRNA, RNAiMax reagent, OPTI-MEM, cells) of RNAi transfection mix. The remaining transfection mixture was plated in 6 well plates for protein analysis. Final concentration of siRNA was 50 nM. When two siRNA were combined, final concentration of siRNA was 25 nM each. For *DR4/5* siRNA experiments, ONC201 or GST-TRAIL was added to the 96 well plates at 48 h post-transfection and MTS reagent was added at 5 days post-transfection for viability measurements. Cells plated in the 6 well plates were harvested for protein analysis at 7 days post-transfection. For *Tfam* siRNA experiments, cell viability, and ATP assays were performed 3 days post-transfection, and cells in 6 well plates were harvested for protein analysis at 3 days post-transfection. siNeg (AllStars Negative Control siRNA, cat#SI03650318), siDR4 (Hs_TNFRSF10A_1 FlexiTube siRNA, cat# SI00056728), siDR5 (Hs_TNFRSF10B_6 FlexiTube siRNA, cat# SI03094063), siTfam #2 (Hs_TFAM2 FlexiTube siRNA, cat# SI00049007), siTfam #4 (Hs_TFAM_4 FlexiTube siRNA, cat# SI00049021), siTfam #10 (Hs_TFAM_10 FlexiTube siRNA, cat# SI04988487) were obtained from Qiagen (Hilden, Germany). For target sequence of siRNA, see Supplementary Table 2.

### Western blotting

Protein was extracted from cells with lysis buffer (1% Triton-X 100, 10mM Tris-HCl, 150mM NaCl, 5mM EDTA, 10% glycerol, 1mM sodium vanadate, 1x cOmplete mini protease inhibitor cocktail [cat#11852700, Roche Diagnostics, Mannheim, Germany]). Protein lysates were cleared of debris by centrifugation at 15,000 ×g for 10 minutes at 4°C, and concentration was assessed by Bio-Rad colorimetric assay (cat#500-0006, Bio-Rad, Hercules, CA). Protein samples were boiled in an equal volume of Laemmli sample buffer (cat#161-0737, Bio-Rad, Hercules, CA, USA) containing 5% β-mercaptoethanol, fractionated by 10% or 4-20% sodium dodecyl sulfate polyacrylamide gel electrophoresis (SDS-PAGE) gel, and transferred to polyvinylidene fluoride membranes (cat#IPVH00010, Millipore, Bedford, MA). Antibodies used in the study were listed in Supplementary Table 2.

### CellTiter-Glo® 2.0 assay (*ATP assay*)

Cells were seeded at 10,000 cells/well in a 96 well white assay plate on the day before treatment. Cells were then treated in RPMI no-glucose growth medium (cat#11879-200, ThermoFisher) supplemented with either glucose (final 10mM) or galactose (final 10mM) with various concentration of the drugs such as ONC201, oligomycin, or metformin. Twenty-four or 72h after treatment, cellular ATP level was measured by adding same volume of CellTiter-Glo® 2.0 Assay reagent (cat#G9242, Promega). All ATP measurements were performed in triplicates and each experiment was carried out at least 3 times. Results were given as the mean +/− the SEM for at least 3 independent experiments.

### Mitochondrial respiration assay

Oxygen Consumption Rate (OCR) and Extra Cellular Acidification Rate (ECAR) were measured with Seahorse XF^e^24 Extracellular Flux Analyzer (Agilent Technologies, Santa Clara, CA, USA) with FluxPaks Mini (cat#100867-100). To measure OCR and ECAR of cells treated with ONC201 for 24 h, cells were seeded on XF24 cell culture microplate (60,000 cells/well for MB231, 30,000 cells/well for SKBR3 cells, 20,000 cells/well for HFF cells) with respective growth medium (Figure 3A, 6G, 7G). Next day, medium was replaced with either glucose (10mM) or galactose (10mM)-supplemented medium with ONC201 (5 μM) or DMSO (Ctl.) and cells were incubated for 24 h. On the day of XF assay, cell culture medium was replaced with XF base medium (cat#102353-100, no glucose, no bicarbonate, no glutamine/GlutaMAX, nor sodium pyruvate) supplemented with glucose or galactose (10mM) with ONC201 (5μM) or DMSO (Ctl.) and subsequently OCR and ECAR were measured 3 times.

For time-course experiments (Figure 3B), MB231 cells were seeded on an XF24 cell culture microplate (60,000 cells/well). The next day, the culture medium was replaced with RPMI medium supplemented with galactose (10mM) and cells were incubated for 24 h. On the day of assay, medium was replaced with XF base medium supplemented with galactose (10mM), and drugs (ONC201 [5μM], oligomycin [1μM], metformin [20mM] and DMSO as control) were administered via injection port. OCR and ECAR were measured every 11 minutes (mix 3 minutes, wait 5 minutes, measure 3 minutes) for 20 h.

For XF analyzer experiments with permeabilized MB231 cells (Supplementary Figure 3E), cells were seeded on an XF24 cell culture microplate (60,000 cells) one day before experiment. On the day of the experiment, 1x mitochondrial assay solution (MAS) (mannitol 220mM, sucrose 103.3mM, KH_2_PO_4_ 10mM, MgCl_2_ 5mM, HEPES 2mM, EGTA 1mM, fatty acid free BSA 0.2%, pyruvate 10mM, malate 1mM, pH=7.2) was prepared. Cells were washed with 1x MAS once, then permeabilized with 1x MAS supplemented with ADP (4mM final) and plasma membrane permeabilizer (PMP, 10μM, cat#102504-100, Agilent). Drugs (ONC201, oligomycin, rotenone etc.) were diluted in 1x MAS (without ADP and PMP) at 10x concentration and administered via ports of a cartridge at 1:9 volume ratio to achieve 1x concentration after administration.

For XF analyzer experiments with freshly isolated mitochondria (Figure 3C), mitochondria were isolated from MB231 cells using mitochondria/cytosol fractionation kit (cat# MIT1000, Millipore), as recommended by the manufacturer's protocol. Briefly, MB231 cells were grown in four or five 15 cm dishes until they became nearly confluent. The mitochondrial fraction was separated from cytosol fraction, and a part of mitochondrial fraction was lysed with the supplied mitochondrial lysis buffer to determine protein concentration. The rest of the isolated mitochondria were re-suspended in 1x MAS supplemented with pyruvate/malate/ADP (pH=7.2) at 20μg/50μl concentration. Isolated mitochondria were plated on an XF24 cell culture microplate at 20μg/50μl/well, the dish was spun down at 3000 x *g*, at 4°C for 15 minutes to let mitochondria attach to the bottom, and the final volume of each well was adjusted to 500 μl by adding 450 μl of 1xMAS supplemented with pyruvate/malate/ADP (pH=7.2). After 3 measurements of basal OCR/ECAR, drugs (DMSO control, Rotenone [final 2μM], ONC201 [final 20μM], and oligomycin [final 2μM]) were added to mitochondria, and OCR/ECAR were measured every 2.5 minutes (mix 30 seconds, wait 0, measure 2 minutes).

For kidney cancer cells, OCR and ECAR were measured using XF96 extracellular flux analyzer (Figure 7A). Twelve thousand cells were added per well in rho0 medium, and the cells were grown for 24 hours. One hour before the assay, the medium was replaced with Seahorse Assay Medium (DMEM without bicarbonate, 25mM D-glucose, 2mM L-glutamine, 1mM sodium pyruvate, pH=7.4).

### Time-lapse live cell imaging

MB231 cells were seeded (60,000 cells/well) on a 24 well glass bottom plates (Part#P24-1.5H-N, CellVis, Mountain View, CA, USA) with RPMI1640 growth medium. The next day, medium was replaced with RPMI1640/galactose (10mM) supplemented with either ONC201 (5 μM) or DMSO, or RPMI1640/glucose (10mM) with GST-TRAIL (200 ng/ml). Each treatment was performed in duplicated wells. The phase-contrast images were captured from each well every 30minutes with AxioVision V4.8.2.0 software and Zeiss Observer Z1 microscope (40x objective) in 37°C, 5% CO_2_ condition for up to 20 h. After recording the data, the image files were compiled using ImageJ (National Institutes of Health). Time-lapse live cell imaging experiments were repeated 4 times.

### Mitochondrial fission analysis

MB231 cells (100,000 cells) were seeded on a 40mm glass cover slip (cat#40-1313-03192, Bioptechs Inc., Butler, PA) placed in a 60 mm cell culture dish. After treatment with ONC201 (5 μM) for 24 h, cells were stained with MitoTracker Deep Red (final 500nM, cat#M22426, ThermoFisher) and Hoechst (1:2000, cat#33342, ThermoFisher) in complete growth medium for 30 minutes at 37°C in a CO_2_ incubator. After washing with complete medium, images were acquired (see below “Confocal live cell imaging and image analysis”).

### Mitochondrial membrane potential analysis

MB231 cells (100,000 cells) were seeded on a 40mm glass cover slip placed in a 60 mm cell culture dish. The next day, cells were co-stained with Tetramethylrhodamine methyl ester (TMRM, cat #T668, ThermoFisher) and MitotTacker Green FM (cat# M7514, ThermoFisher) for 30 minutes at 37°C CO_2_ incubator. TMRM binds to lipophilic cations accumulated by mitochondria in proportion to membrane potential (ΔψΨm). MitoTracker Green FM covalently binds to mitochondrial matrix independent of ΔψΨm. After washing cells with complete medium twice, cells were treated with DMSO (Ctl.) or ONC201 (5 μM). Twenty-four hours later, Hoechst was added to culture medium (1:2000) and subsequently cell imaging was performed (see below “Confocal live cell imaging and image analysis”).

### Mitochondrial DNA detection

MB231 cells seeded on a 40 mm glass cover slip as above were treated with DMSO (Ctl.) or ONC201 (5μM). Twenty-four hours later, cells were stained with MitoTracker Deep Red (final 500nM), PicoGreen (1:500, cat# P11495, ThermoFisher) and Hoechst 33342 (1:2000, cat# H3570, ThermoFisher) in complete medium for 30 minutes at 37°C in a CO_2_ incubator. After washing with complete medium, images were acquired as described below.

### Confocal live cell imaging and image analysis

Coverslips were mounted on a temperature controlled, sealed chamber suitable for live cell imaging. Images were acquired with an Olympus FluoView 1000 Confocal Microscope (Olympus America) equipped with a 60x PLAPON oil immersion objective (NA 1.42) preheated to 37°C to avoid thermal aberrations. MitoTracker Green and Pico Green were excited with a 488-nm laser, TMRM was excited with a 561-nm laser, and MitoTracker Deep Red was excited with a 633-nm laser. Z-scans on cells were acquired at scan speeds of either 4 or 8 μs/pixel (320 ×320 pixels). Images were stored as TIFF files and analyzed using ImageJ (National Institutes of Health) or Imaris (Bitplane) software. Each experiment was performed in duplicates, and at least 3-4 images were captured from each sample.

### Electron microscopy

MB231, HFF, and T47D cells were seeded in 6-well plates (200,000 cells/2 ml/well) with RPMI1640 growth medium and treated with ONC201 (5μM). After treatment for different times, cells were washed with phosphate buffer saline (PBS), trypsinized, and centrifuged at 1,000 rpm (216 x *g*) for 10 minutes. After aspirating the supernatant, fixative (2% glutaraldehyde, 0.1M sodium cacodylate) was added to the cell pellets, samples were incubated for 2h at room temperature, then stored at 4°C. Transmission electron microscope (TEM) images were processed by Electron Microscopy Laboratory (EML) in the Advanced Technology Research Facility (ATRF), Leidos Biomedical Research, Inc. in Frederick National Laboratory for Cancer Research (Frederick, MD, USA).

### Mitochondrial DNA copy number analysis (experiments shown in Figure 5B, 5C, 6F, Supplementary Figure 5, Supplementary Figure 8J)

Genomic DNA from cells were isolated with DNeasy Blood & Tissue kit (cat#69504, Qiagen). Change of mtDNA copy number was analyzed by quantitative PCR (qPCR) with Human mitochondrial to nuclear DNA ratio kit (cat#7246, Takara Bio USA Inc., Mountain View, CA). Briefly, relative mtDNA copy number was obtained by qPCR of two mtDNA genes (ND1, ND5) and two nuclear DNA (nDNA) genes (SLCO2B1, SERPINA1). For PCR reaction, PowerUp SYBR Green Master Mix was used (cat#A25742, ThermoFisher) with ViiA™ 7 Real-Time PCR System with 384-well block (cat#4453536, ThermoFisher). PCR cycle conditions were 50°C (2 minutes), 95°C (2 minutes), followed by 40 cycles of two-step cycling of 95°C (15 seconds) and 60°C (60 seconds). C*t* value of each target gene was obtained from qPCR results, then ΔC*t_1_*(C*t* [ND1]- C*t* [SLCO2B1]) and ΔC*t_2_*(C*t* [ND5]-C*t* [SERPINA1]) were obtained for each time point sample. Average value of 2^ΔC*t1*^ and 2^ΔC*t2*^ was calculated as relative mtDNA copy number.

### Generation of rho0 cells

MB231^*^ rho0 (mtDNA depleted) cell lines were established by EtBr treatment, according to previously published protocol [48]. Briefly, cells were maintained in rho0 medium (DMEM high glucose [cat#11965092, ThermoFisher, 25mM glucose, 4mM L-glutamine] supplemented with 10 % FBS and 1 % P/S, sodium pyruvate [final 100ug/ml], uridine [final 50ug/ml]). Cells were plated at 1x 10^5^ cells/10 cm and treated with EtBr (final 50 ng/ml). Approximately 1 week later, majority of the cells in the culture became non-viable and detached from the dish. Following several medium changes over the next few days, the remaining cells were trypsinized and re-plated onto several new 10 cm dishes at differing dilutions, and colonies were formed for approximately 2 weeks. Single colonies were picked using cloning rings and expanded further in the absence of EtBr. UOK121^*^ rho0 cell lines were established by EtBr treatment of UOK121 (renal cell carcinoma)[66] using similar protocol describe above. In brief, 1 × 10^6^ UOK121 cells were plated on a 15cm tissue culture dish and grown continuously in the rho0 medium and viable single colonies were isolated and expanded as described above. Depletion of mtDNA was confirmed by qPCR using t-RNA-Leu (mtDNA gene) and POLG (DNA polymerase subunit gamma-1, nDNA gene, for primer sequence see Supplementary Table 2). The same PCR cycling condition was used as noted in the section above. Relative mtDNA copy number of parent and rho0 cells (Figure 7C, 7F) was obtained as 2^ΔC*t*(C*t*[t-RNA-Leu]-C*t*[POLG])^. MB231^*^ rho0 cell clonal lines, UOK262, UOK121^*^ rho0 clonal line as well as parent cell lines were all maintained with rho0 medium.

### RNA extraction for RNAseq

MB231 cells were seeded on four 6 well plates (1.25 × 10^6^ cells/well). Cells in each plate were treated with ONC201 (5 μM) for different times (0, 3 h, 6 h, 24 h, 48 h). Cells were trypsinized and centrifuged at 310 x *g* for 5 minutes, then transferred to 1.5 ml tube, washed with PBS twice, snap frozen with ethanol-dry ice bath. RNA was isolated from cells using TRIZOL reagent (cat#15596018, ThermoFisher) as recommended by the manufacturer.

### Library preparation and illumina sequencing

One microgram of total RNA per each time point was used as the input to an mRNA capture with oligo-dT coated magnetic beads. The mRNA was fragmented, and then a random-primed cDNA synthesis was performed. The resulting double-strand cDNA was used as the input to a standard Illumina library prep (TruSeq Stranded mRNA LT library prep kit, Illumina) with end-repair, adapter ligation and PCR amplification being performed to generate the library. The indexed mRNAseq libraries were quantitated by qPCR, pooled with equimolar amounts and sequenced on an Illumina HiSeq 2500 sequencer for a 2 x125 cycle run.

### Reverse transcriptase-PCR

For experiment shown in Supplementary Figure 1B, MB231, MB468, SKBR3, T47D cells were grown in 60mm culture dishes, then treated with ONC201 at 5μM for 0, 24, 48, 72h. Total RNA was isolated with TRIZOL as described above. One microgram total RNA/each cell line was used for RT reaction (42°C for 15 minutes, 95°C for 3 minutes) with QuantiTect Reverse Transcription Kit (cat#205311, Qiagen). PCR reaction was performed with 2x PowerUp SYBER Green master mix (cat#A25777, ThermoFisher Scientific). Primers used for qPCR were: TRAIL (Hs_TNFSF10_1_SG [cat#QT00079212]), DR4 (Hs_TNFRSF10A_1_SG [cat#QT00065723]), DR5 (Hs_TNFRSF10B_1_SG [cat#QT00082768)]), TFAM (Hs_TFAM_1_SG [cat#QT00012782]), DDIT3/CHOP (Hs_DDIT3_1_SG [cat# QT00082278]), GAPDH (Hs_GAPDH_1_SG [cat#QT00079247]), from Qiagen. All qPCR was performed with three technical replicates. PCR cycle condition was 50°C (2 minutes), 95°C (2 minutes), followed by 40 cycles of two-step cycling of 95°C (15 seconds) and 60°C (60 seconds). C*t* value of each target gene was obtained from qPCR results. Results were quantitatively analyzed with ΔΔC*t* method. Namely, ΔΔC*t* = (C*t* (target, treated) − C*t* (GAPDH, treated)) − (C*t* (target, untreated) − C*t* (GAPDH, untreated)). Then 2-ΔΔC*t* was used as relative copy number of each transcript.

For experiment shown in Supplementary Figure 9, total RNA was isolated and cDNA was prepared as described above. PCR reactions were performed with 2x PowerUp (described above) with DRD2 primers (Hs_DRD2_1_SG Quantitect Primer Assay, cat# 249900-QT00012558) and actin primers (Hs_ACTB_1_SG Quantitect Primer Assay, cat#249900-QT00095431, Qiagen). PCR cycle condition was 50°C (2 minutes), 95°C (2 minutes), followed by 40 cycles of two-step cycling of 95°C (15 seconds) and 60°C (60 seconds). C*t* value of each target gene was obtained from qPCR results. For quantitative analysis, first, ΔC*t* (DRD2 C*t* –actin C*t*) was obtained in each cell line, ΔΔC*t* (difference of ΔC*t* between each cell line and reference cell line (GSC)) was calculated. Then 2-ΔΔC*t* was used as relative copy number of DRD2 transcript.

### Bioinformatics

Bioinformatics analysis of RNAseq data was performed by CCR Collaborative Bioinformatics Resource, Leidos Biomedical Research, Inc. To generate heat map, 125 bp paired end reads for each sample were aligned using 2-pass STAR [67] aligner (v2.5.3a) to the human reference genome, hg19. Reads aligning to gene annotations (gencode release 19) were counted using RSEM [68] (v1.3) and differential gene expression analysis was performed using DESeq2 [69] R package. log2 fold changes for 3, 6, 12 and 24 h time points were then extracted for different time points and hierarchical clustering was performed to visualize expression changes for a variety of gene-sets as heatmaps generated using ClustViz [70]. Pre-ranked differentially expressed genes from DESeq2 [69] output where used to perform Gene Set Enrichment Analysis (GSEA) [71] with the Molecular Signature Database (MSigDB) [71] gene-set collection. Normalized enrichment scores (NES) for all mitochondrially relevant gene-sets for all time points where extracted from the GSEA results and hierarchical clustering was performed to depict temporal changes in the enrichment patterns.

### CellMiner

Data analysis of ONC201 on the NCI-60 was performed using the NCI CellMiner website (http://discover.nci.nih.gov/cellminercdb).[72, 73] Drug activities were plotted as z-score and *TFAM* mRNA expression as Affymetrix units.

### Statistics

The significance of differences in data was determined with Student's *t*-test, or two-way ANOVA (see more details in figure legends). The differences were considered significant when *p* value was less than 0.05.

Disclaimer (Electron Microscope Laboratory, Leidos Biomedical Research, Inc. Frederick National Laboratory for Cancer Research): This project has been funded in whole or in part with federal funds from the National Cancer Institute, National Institutes of Health, under contract HHSN26120080001E. The content of this publication does not necessarily reflect the views or policies of the Department of Health and Human Services, nor does mention of trade names, commercial products, or organizations imply endorsement by the U.S. Government.

## SUPPLEMENTARY MATERIALS FIGURES AND TABLES









## Abbreviations

AMPK: 5′-AMP-activated protein kinase
ATF4: Activating Transcription Factor 4
ATP: Adenosine triphosphate
CHOP: C/EBP-homologous protein
DMSO: Dimethyl sulfoxide
DR4/5: death receptor 4/5
ECAR: extra cellular acidification rate
ER: endoplasmic reticulum
EtBr: ethidium bromide
FH: fumarate hydratase
Foxo3a: Forkhead box protein O3
GST: glutathione S-transferase
HFF: human foreskin fibroblast
HER2: human epidermal growth factor receptor 2
ISR: integrated stress response
mtDNA: mitochondrial deoxyribonucleic acid
NEM: nuclear encoded mitochondrial
OCR: oxygen consumption rate
OxPhos: oxidative phosphorylation
PBS: phosphate buffer saline
POLG: DNA polymerase subunit gamma-1
p70S6K: ribosomal protein S6 kinase beta-1
qPCR: quantitative polymerase chain reaction
Tfam: mitochondrial transcription factor A
TNBC: triple negative breast cancer
TRAIL: Tumor Necrosis Factor-related apoptosis-inducing ligand
TEM: transmission electron microscopy
UPR: unfolded protein response
Z-VAD-FMK: carbobenzoxy-valyl-alanyl-aspartyl-[O-methyl]-fluoromethylketone.
