# Supplementary material for: ONC201 kills breast cancer cells in vitro by targeting mitochondria
Source: Oncotarget. 2018 Apr 6;9(26):18454–79. doi: 10.18632/oncotarget.24862 (PMC5915085; doi:10.18632/oncotarget.24862)
Supplement: Supplementary file 1 [file oncotarget-09-18454-s001.pdf]

ONC201 kills breast cancer cells *in vitro* by targeting mitochondria

SUPPLEMENTARY MATERIALS

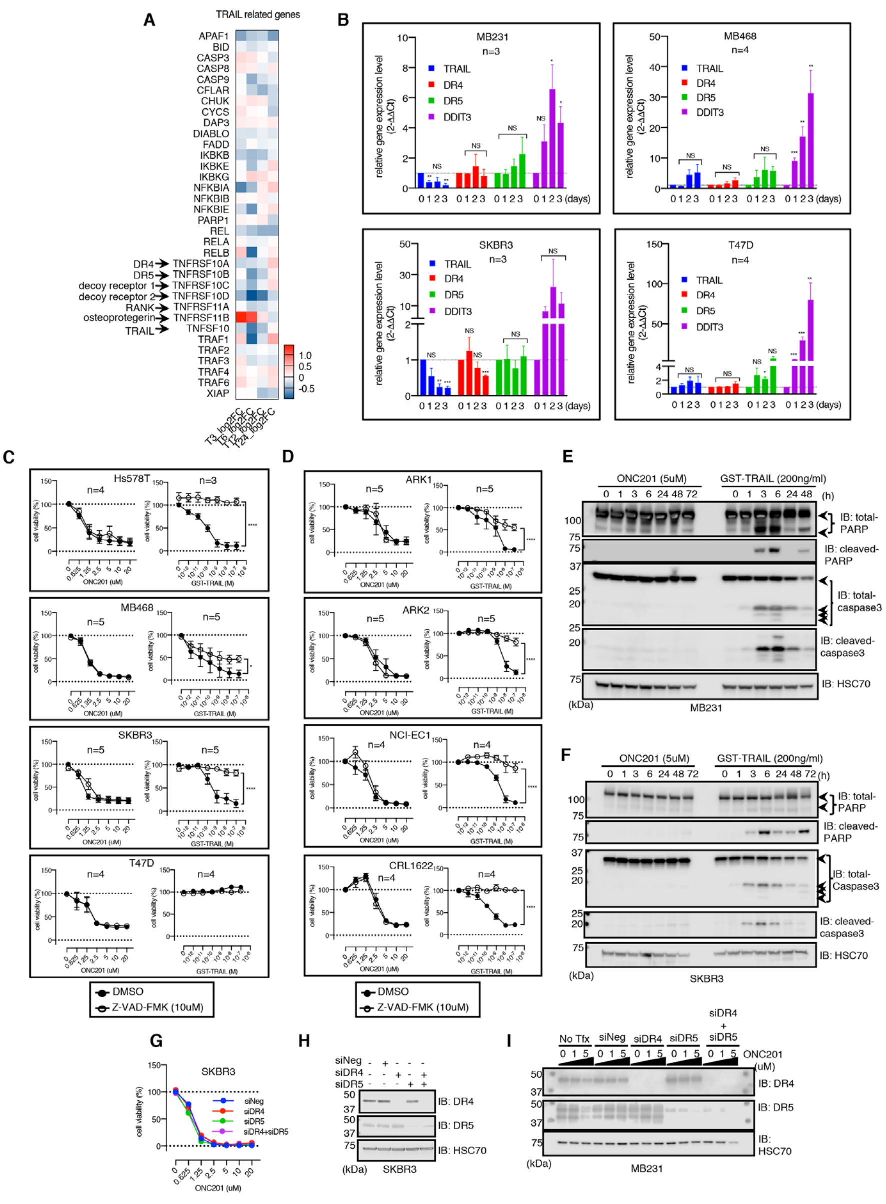

Supplementary Figure 1: ONC201 decreases cell viability in multiple breast and endometrial cancer cells lines via non-caspase dependent mechanism. (A) Heat map showing fold changes (Log2) of TRAIL-related genes at 3, 6, 12, 24 h ONC201 (5μM) treatment compared to no (0 h) treatment (RNAseq) in the MB231 cell line. **(B)** Time dependent effects of ONC201 (5μM) on TRAIL, DR4, DR5, and DDIT3 mRNA by qPCR were examined in MB231, MB468, SKBR3, and T47D cell lines. Four bars for each transcript represent 0, 1, 2, 3 days of ONC201 treatment. Data is shown as mean +/- SEM of multiple independent experiments normalized to GAPDH mRNA. NS= not significant, *p<0.05, **p<0.01, ***p<0.001 compared to each time zero (*t*-test). **(C and D)** MTS assays in various breast cancer cell lines (C) and endometrial cancer cells lines (D) treated with ONC201 or GST-TRAIL for 5 days in the presence or absence of Z-VAD-FMK (10μM). Results are shown as mean +/- SEM of multiple experiments. *p<0.05, ****p<0.0001, two-way ANOVA. **(E and F)** Western blot showing the effect of ONC201 or GST-TRAIL on apoptosis markers in MB231 (E) and SKBR3 (F). **(G)** MTS assay of SKBR3 cells transfected with siRNA, followed by ONC201 for 5 days. **(H)** Western blot confirming siRNA-induced knockdown of DR4 and DR5 in SKBR3 cells. **(I)** Verification that ONC201 does not upregulate endogenous DR4/DR5 expression after siDR4 and siDR5 transfection (MB231). Cell lysates were collected 3 days after ONC201 treatment following 2 days of siRNA transfection.

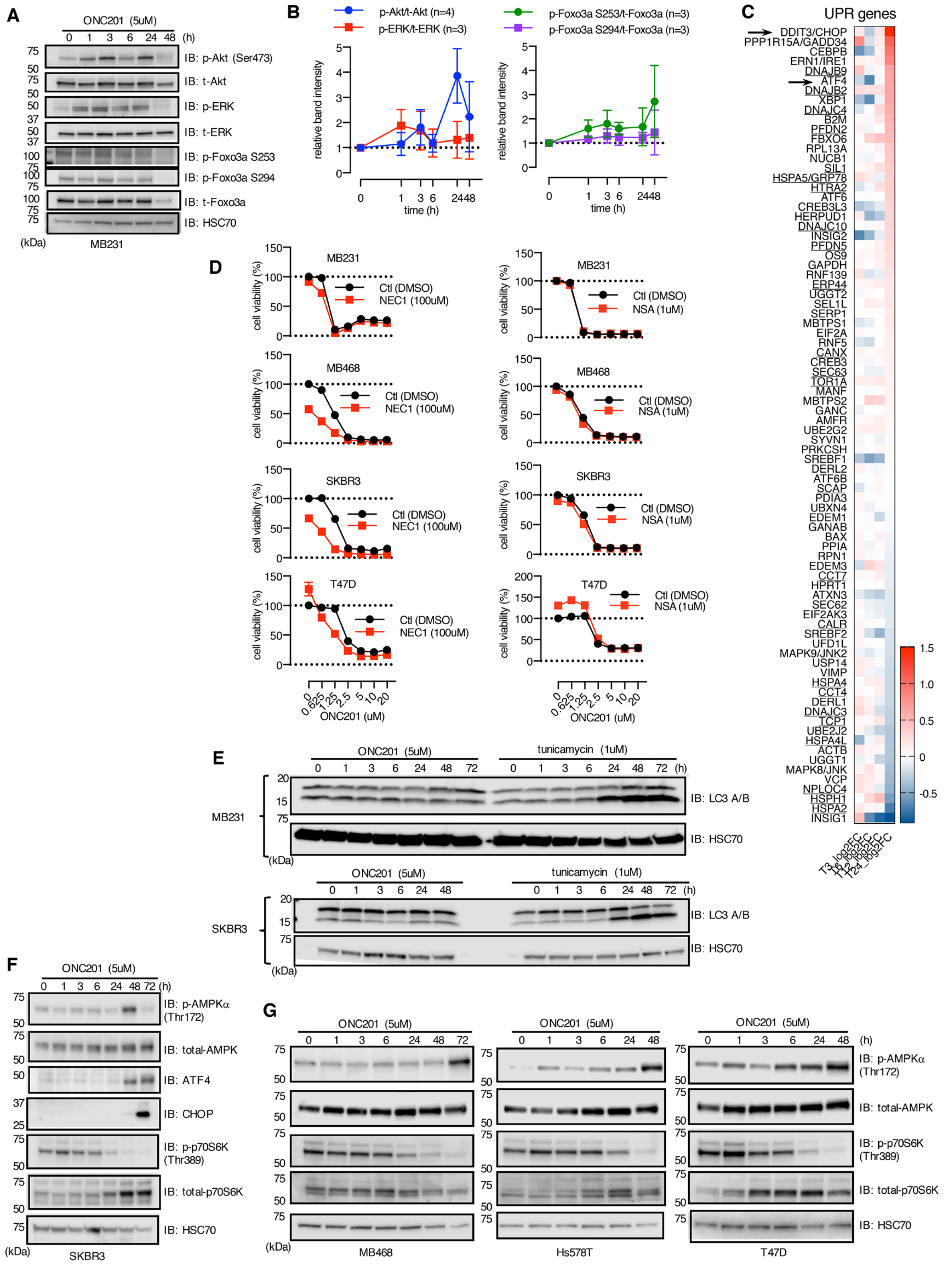

Supplementary Figure 2: ONC201 stimulated AMPK activation and downregulated p70S6K across different subtypes of breast cancer cell lines. **(A)** Western blot showing time-dependent effect of ONC201 (5μM) on Akt, ERK, Foxo3a in MB231 cells. **(B)** Quantitative analysis of phosphorylation status of Akt, ERK and Foxo3a in MB231 cells treated with ONC201. Data is shown as mean +/- SEM of multiple independent experiments. **(C)** Heat map showing fold changes (Log2) of unfolded protein response genes at 3, 6, 12, 24 h ONC201 (5μM) treatment compared to no (0 h) treatment (RNAseq). Genes underlined are chaperones. Arrows indicate CHOP and ATF4. **(D)** MTS assays after 5 days treatment with ONC201 in the presence of necroptosis inhibitors NEC1 (left) or NSA (right) in 4 breast cancer cell lines. **(E)** Western blot showing time-dependent effect of ONC201 or tunicamycin (positive control of ER stress-induced autophagy) on LC3 in MB231 (top panel) and SKBR3 (bottom panel). **(F)** Western blot showing time-dependent effect of ONC201 (5μM) on AMPK, stress proteins, and p70S6K in SKBR3 cells. **(G)** Western blot showing time-dependent effect of ONC201 (5μM) on AMPK and p70S6K in MDA-MB468, Hs578T, and T47D cells.

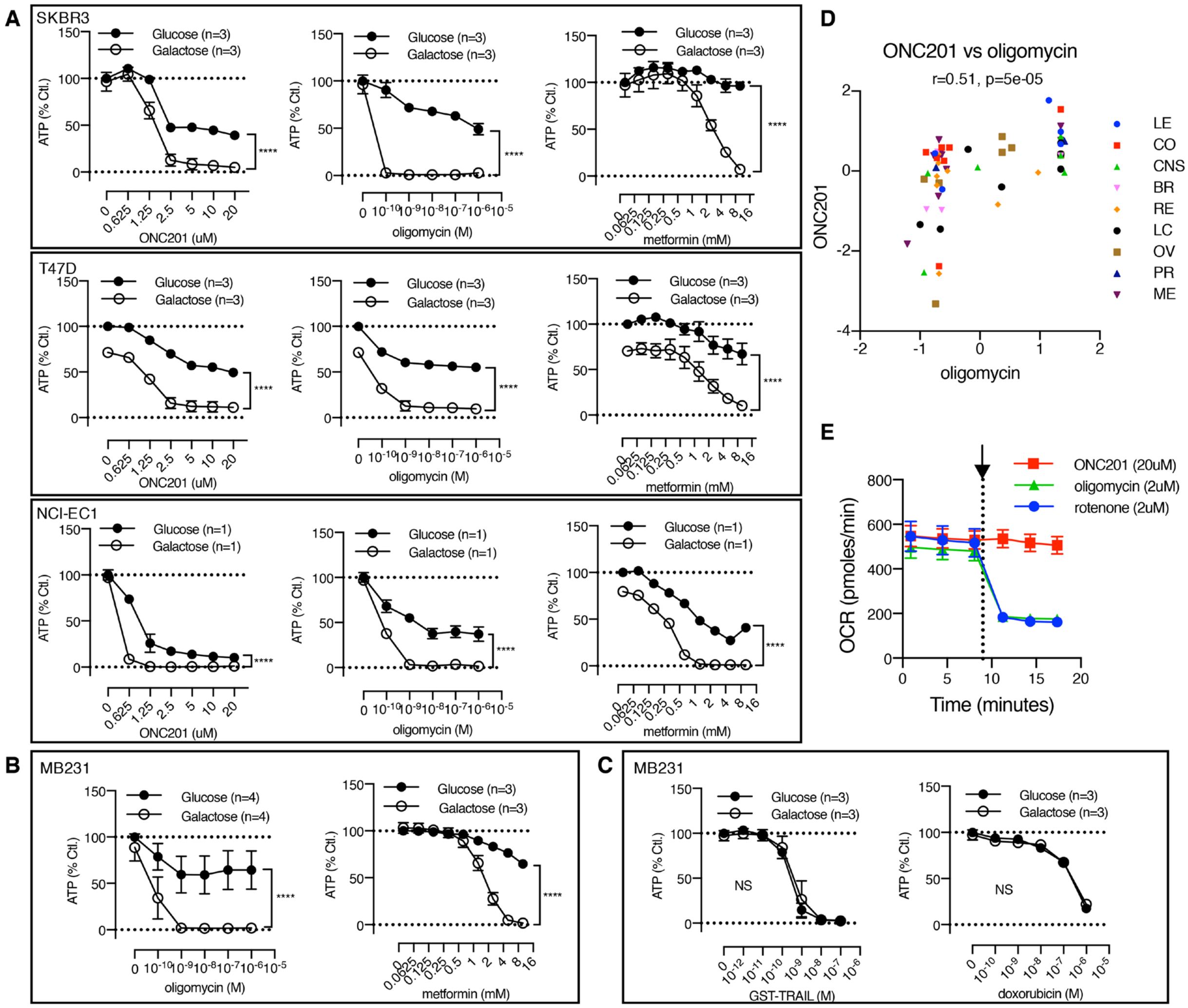

Supplementary Figure 3: Comparison of mitochondrial targeting drugs and ONC201. **(A)** ATP assays after 3 days treatment with ONC201, oligomycin, metformin in glucose or galactose medium. The results are shown as mean +/- SEM of summary of 3 (SKBR3, T47D) and 1 (NCI-EC1) experiments. **(B)** ATP assays after 3 days treatment with oligomycin or metformin, in glucose or galactose medium in MB231 cells. ****p<0.0001, two-way ANOVA. **(C)** ATP assays after 3 days treatment with GST-TRAIL and doxorubicin in MB231 cells. Glucose/galactose does not affect non-mitochondria-targeting drugs. NS=not significant. **(D)** CellMiner data showing correlation between ONC201 activity and cytotoxicity of oligomycin in multiple cancer cell lines. LE (leukemia), CO (colon), CNS (central nervous system), BR (breast), RE (renal), LC (non-small cell lung), OV (ovarian), PR (prostate cancer), ME (melanoma). **(E)** XF analyzer experiment using permeabilized MB231 cells. After 3 measurements of basal OCR, drugs (rotenone, ONC201, oligomycin) were injected via ports, and OCR was measured every ~3 minutes, 3 times. Each drug treatment group had 7 replicates. Data is shown as mean +/-SEM of one representative result of 4 experiments.

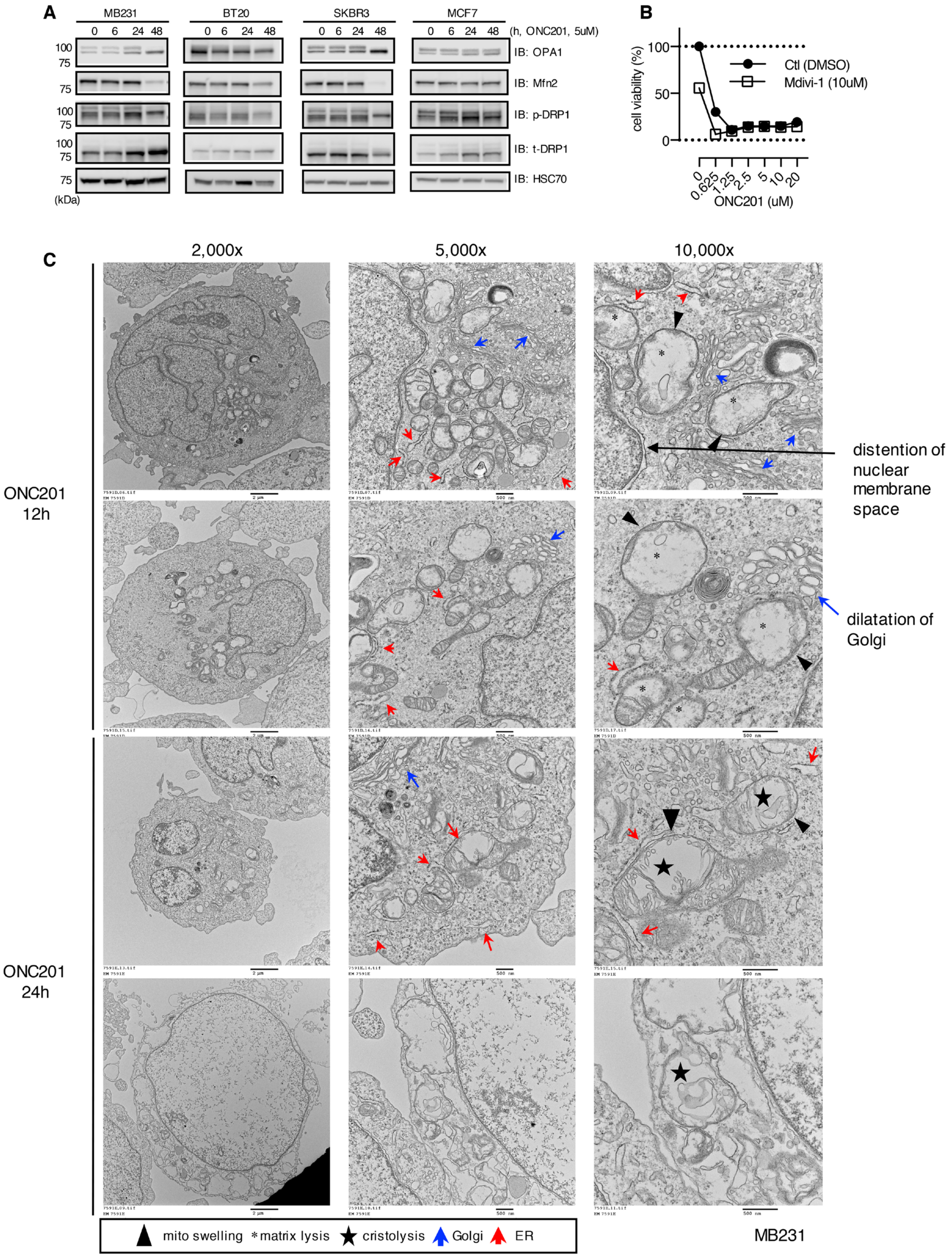

Supplementary Figure 4: ONC201 induces mitochondrial structural damage. **(A)** Western blot showing proteins involved with mitochondrial fusion (OPA1, Mfn2) and fission (DRP1). **(B)** MTS assays after 5 days treatment of ONC201 in the presence or absence of Mdivi-1 in MB231 cells. **(C)** TEM images of MB231 cells treated with ONC201 (5μM) for 12 h and 24 h. Note that Golgi and ER remained relatively intact until later time point compared to mitochondria.

(*Continued*)

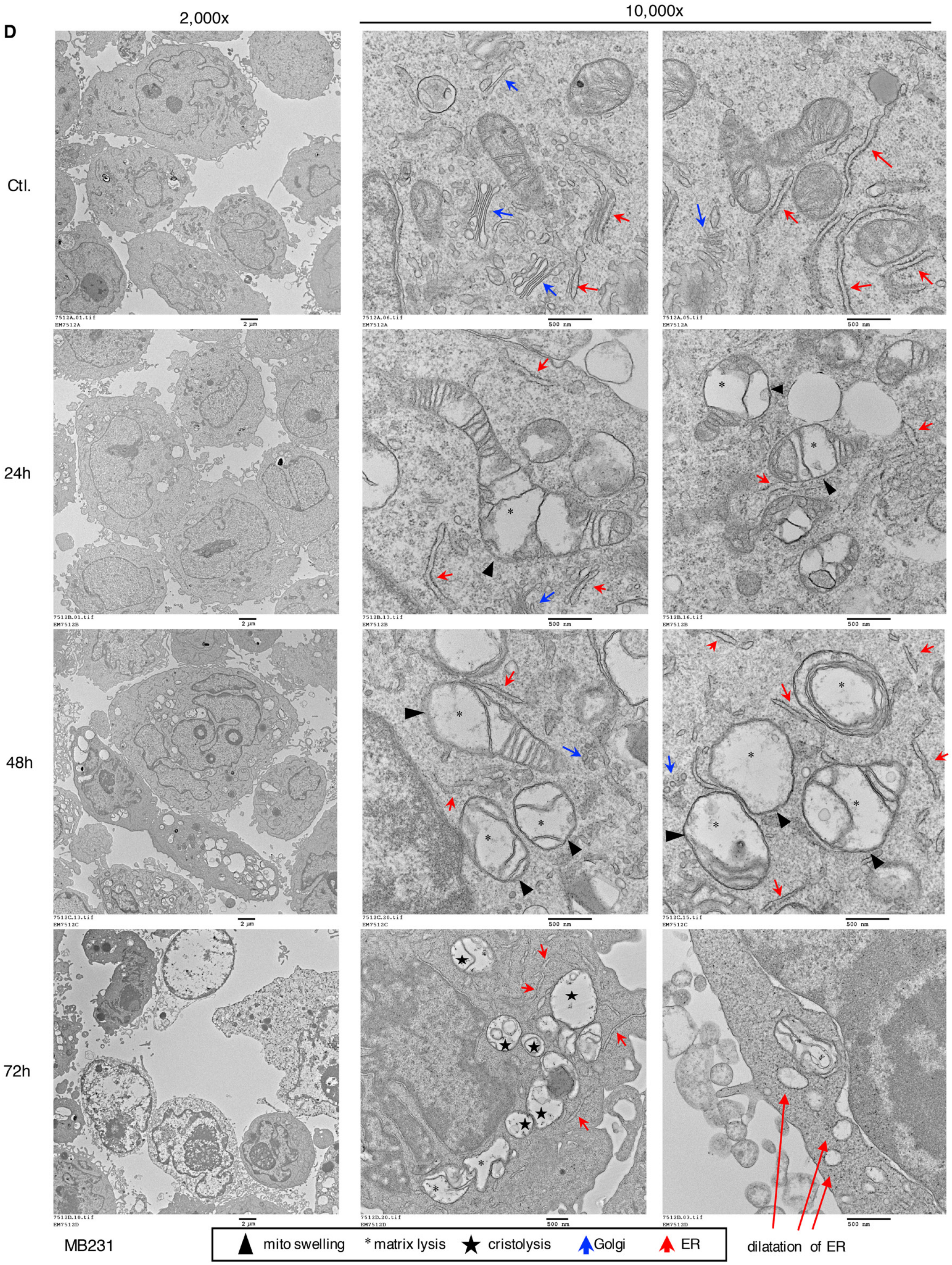

Supplementary Figure 4 (*Continued*): (D) TEM images of MB231 cells treated with ONC201 (5μM) for longer time points (24, 48, 72 h).

(*Continued*)

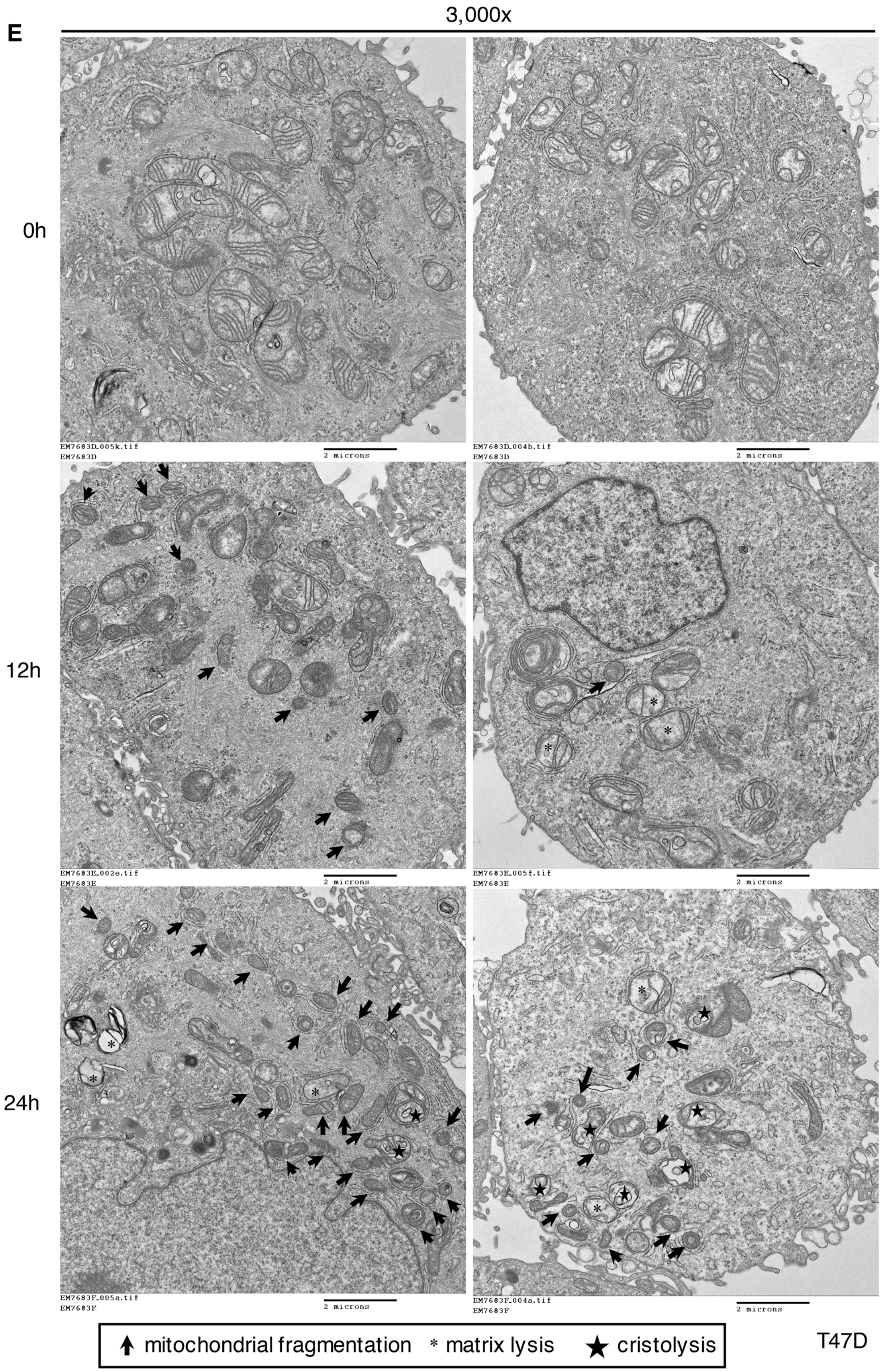

Supplementary Figure 4 (*Continued*): (E) TEM images of T47D cells treated with ONC201 (5μM) for 12 h and 24 h, two representative pictures per each time point.

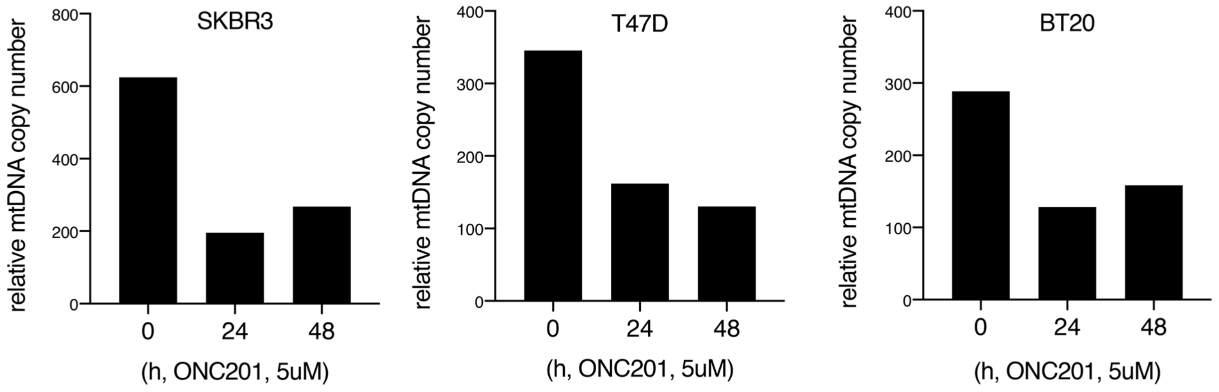

Supplementary Figure 5: ONC201 decreases mtDNA copy number in multiple breast cancer cell lines. Quantitative analysis of relative mtDNA copy number of multiple breast cancer cell lines treated with ONC201 (5μM) for different times in glucose medium.

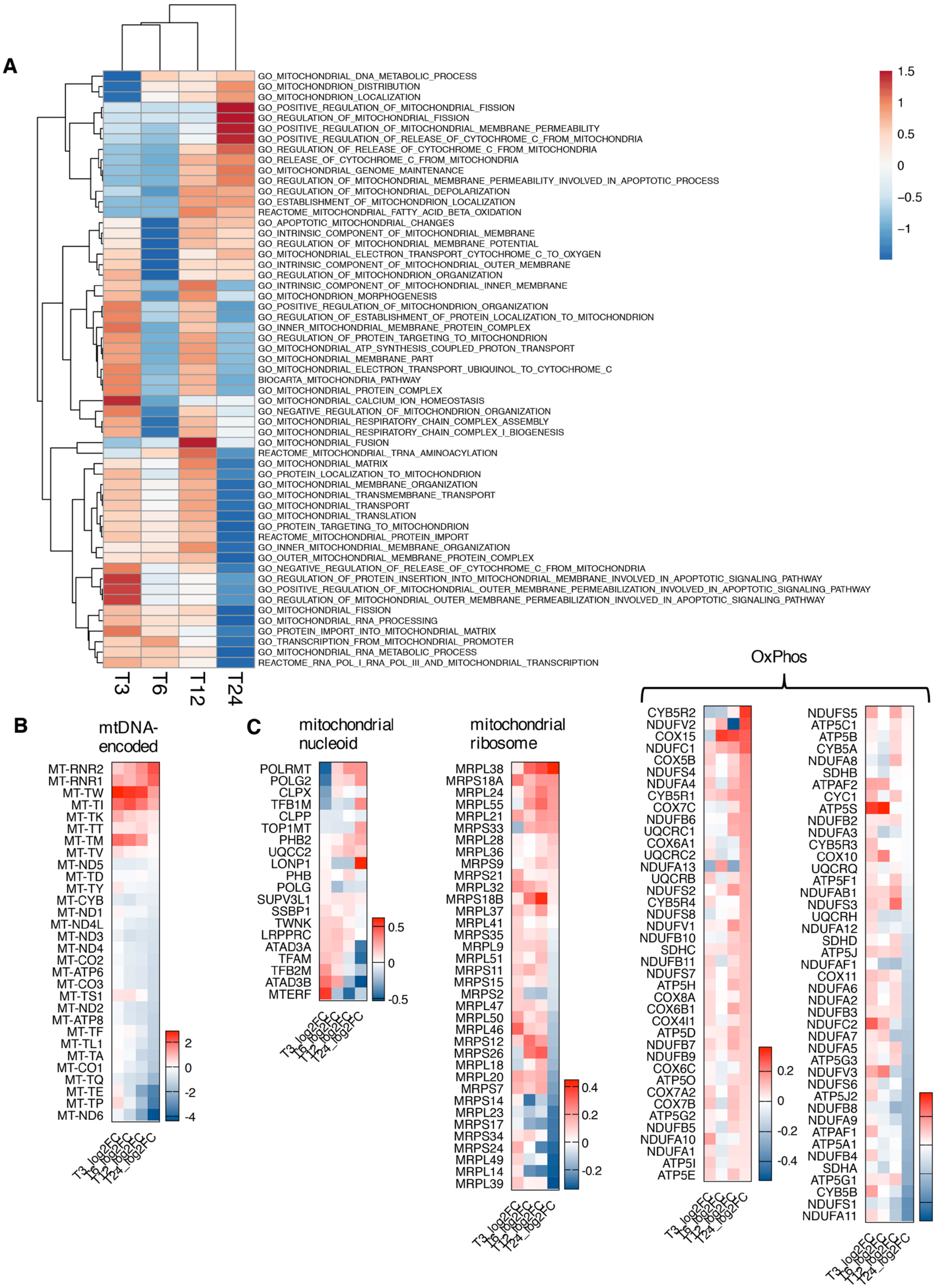

Supplementary Figure 6: RNAseq data supports hypothesis that ONC201 targets mitochondria. (A) GSEA analysis of MB231 cells treated with ONC201 (5μM) for 0, 3, 6, 12, 24 h. **(B)** Heat map showing fold changes (Log2) of mitochondrially-encoded genes at each time point (3, 6, 12, 24 h) compared to no (0 h) treatment (RNAseq). **(C)** Heat map showing fold changes (Log2) of nuclear encoded mitochondrial genes (NEM), such as mitochondrial nucleoid genes, mitochondrial ribosomal genes, and mitochondrial OxPhos genes at each time point (3, 6, 12, 24 h) compared to no (0 h) treatment (RNAseq).

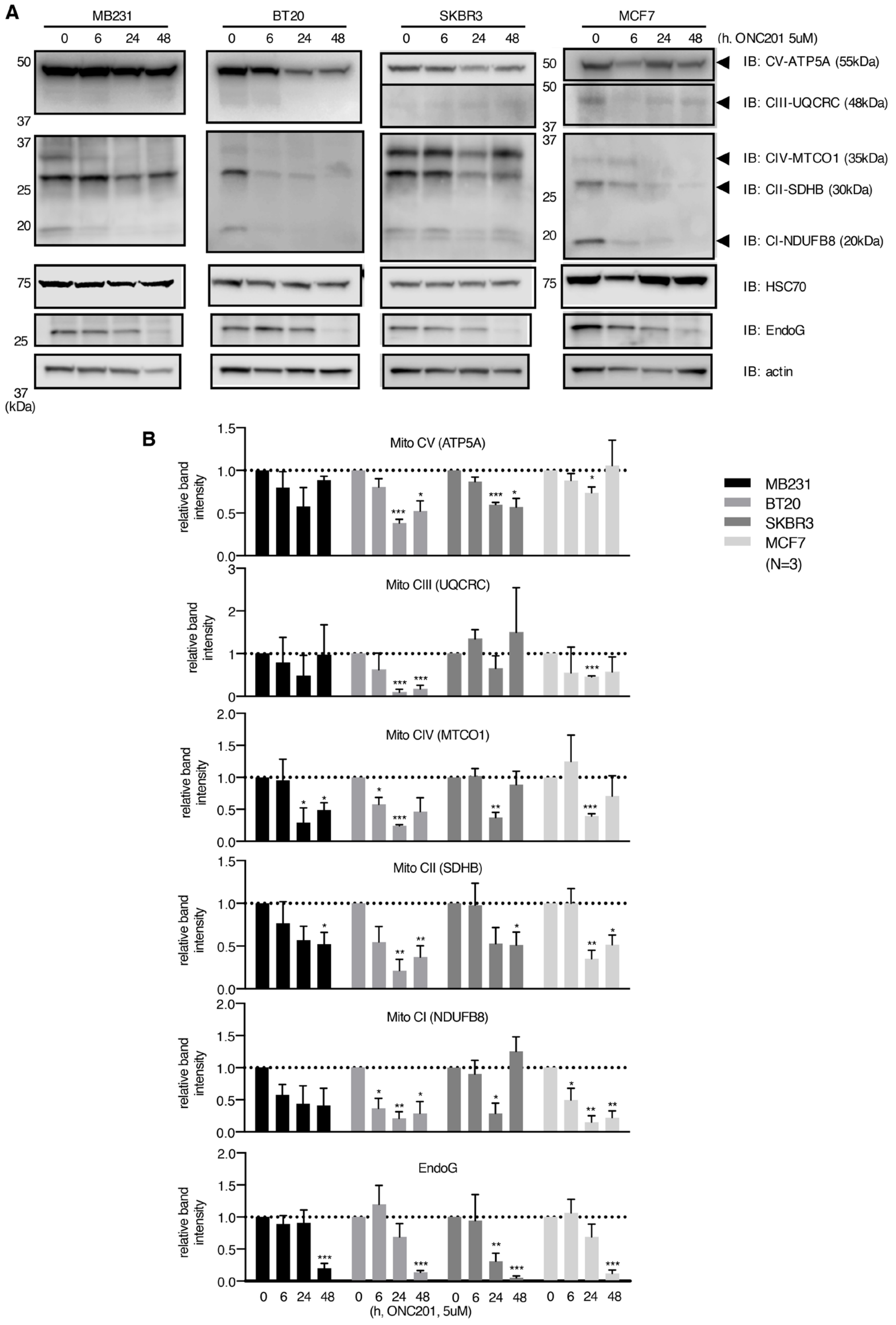

Supplementary Figure 7: ONC201 depletes mitochondrial OxPhos and endonuclease G at protein level. **(A)** Western blot showing impact of ONC201 on mitochondrial OxPhos proteins and endonuclease G (EndoG) in 4 different breast cancer cell lines. Data shown here is one representative result of 3 independent experiments. **(B)** Quantitative analysis of band intensities obtained with Western blot (A). Data is shown as mean +/- SEM of relative band intensity normalized with loading control (HSC70 for OxPhos genes, actin for EndoG) in 3 independent experiments. $^{*}p<0.05$, $^{**}p<0.01$, $^{***}p<0.001$ compared with time zero control (*t*-test).

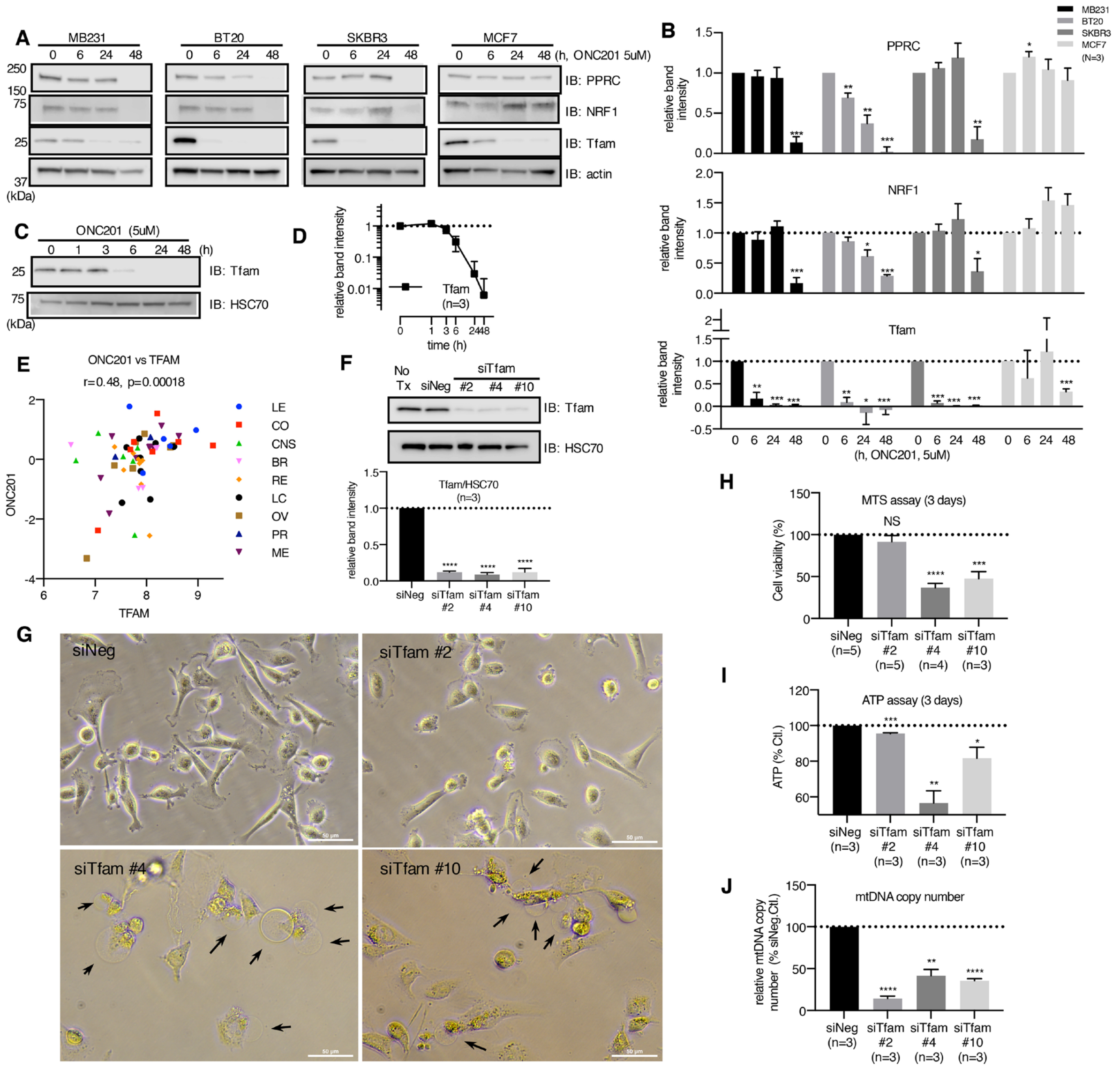

Supplementary Figure 8: ONC201 decreases Tfam, and Tfam knockdown partially mimics ONC201 effect. (A) Western blot showing the effect of ONC201 on mitochondrial biogenesis genes. **(B)** Quantitative analysis of Western blot (A), shown as mean +/- SEM of relative band intensity normalized with actin from 3 independent experiments. *p<0.05, **p<0.01, ***p<0.001 compared to t=0. **(C)** Western blot showing the effect of ONC201 on Tfam in earlier time points (1, 3 h in addition to 6, 24, 48 h, as shown in A) in MB231 cells. **(D)** Relative band intensity of Tfam shown in C. Data is shown as mean +/- SEM of 3 independent experiments. **(E)** CellMiner NCI-60 data showing correlation between ONC201 activity and high expression of *Tfam*. **(F)** Western blot showing Tfam knockdown by siRNA (72 h post-transfection). Top; 1 representative result of 3 experiments. Bottom; relative band intensity of Tfam normalized with HSC70, shown as mean +/- SEM of 3 independent experiments. ****P<0.0001 compared to siNeg (*t*-test). **(G)** Images of MB231 cells transfected with siRNA. Scale bar = 50μm. Arrows indicate plasma membrane ballooning phenotype. **(H)** MTS assay, **(I)** ATP assay, and **(J)** qPCR performed 3 days after siRNA transfection. Data is shown as mean +/- SEM of multiple independent experiments. *p<0.05, **p<0.01, ***p<0.001, ****p<0.0001 compared to siNeg (*t*-test).

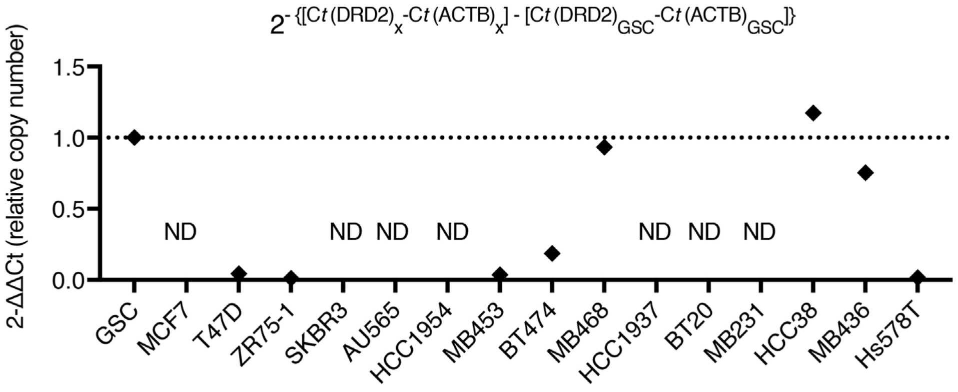

Supplementary Figure 9: Detection of DRD2 expression in breast cancer cell lines by qPCR. Expression of DRD2 transcripts in breast cancer cell line was compared to glioblastoma stem cells (GSC, a positive control) by qPCR. ND = not detected.

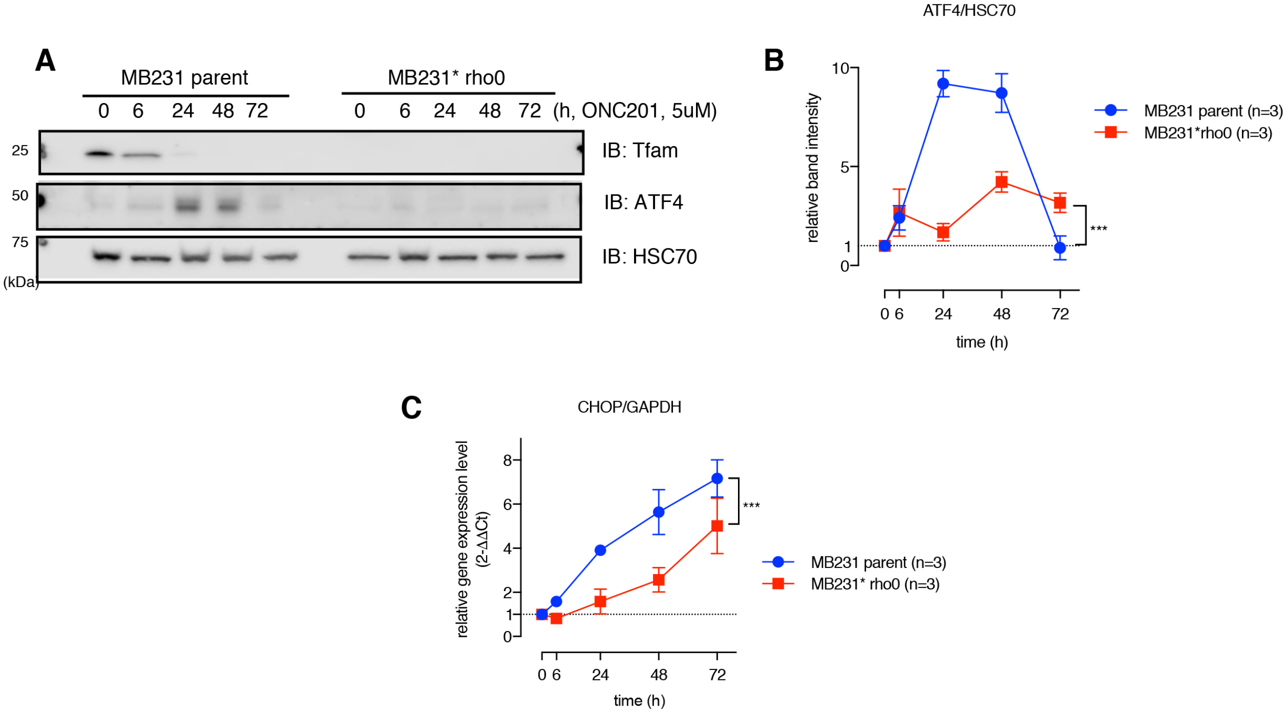

Supplementary Figure 10: The role of functional mitochondria in stress response to ONC201. (A) Immunoblot showing time-dependent induction of ATF4 by ONC201 in MB231 parental and rho0 cells. The panel is one representative result of 3 independent experiments. **(B)** The quantitative analysis of ATF4 induction by ONC201 obtained by band intensities in immunoblotting, normalized with loading control (HSC70). Data shown are the mean +/- SEM of 3 independent experiments. ***$p<0.001$, two-way ANOVA. **(C)** The quantitative analysis of CHOP transcript induced by ONC201, normalized with GAPDH. Data shown are the mean +/- SEM of 3 independent experiments. ***$p<0.001$, two-way ANOVA.

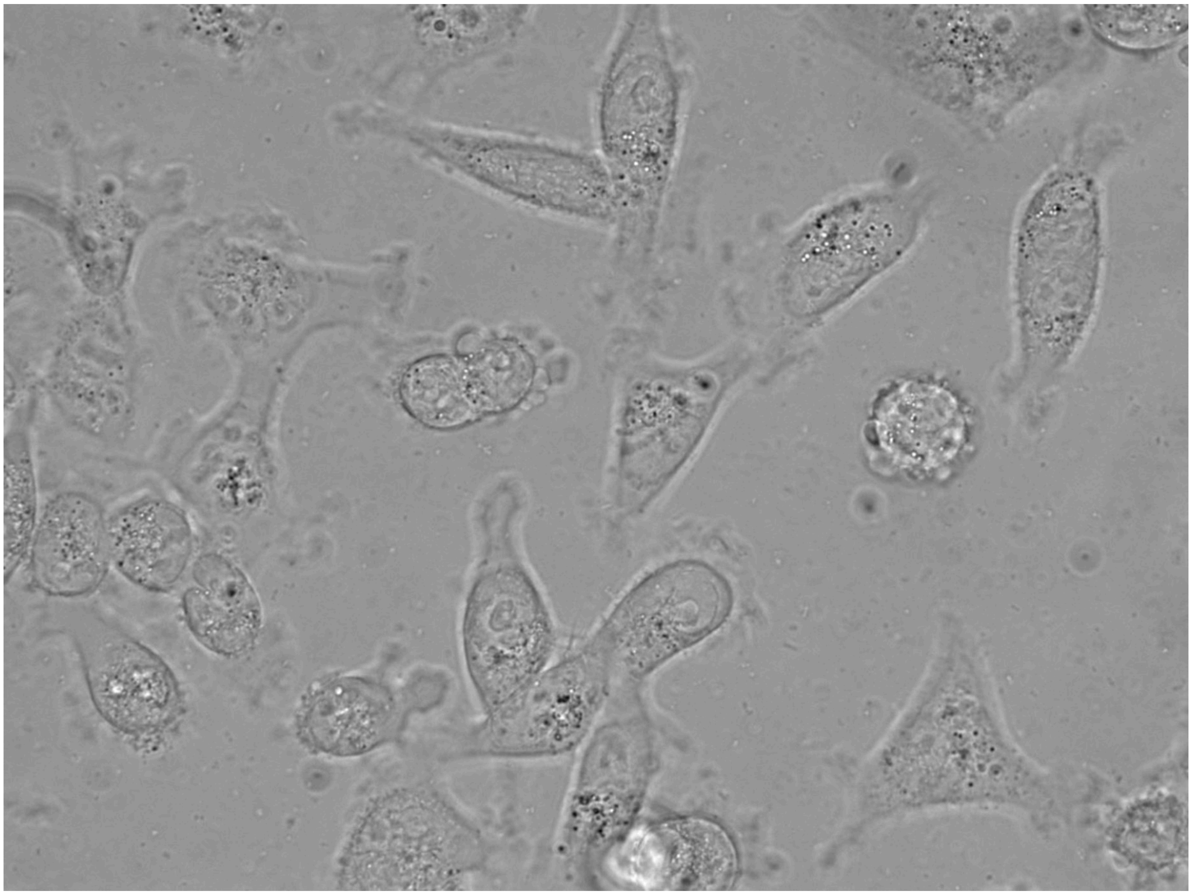

Supplementary Movie 1: Time lapse imaging of MB231 cells treated with DMSO (Ctl.). Cells were treated with DMSO (Ctl.) in RPMI medium supplemented with galactose, and monitored every 20 min with for 20 h. Scale bar= 50µm.

See Supplementary File 1

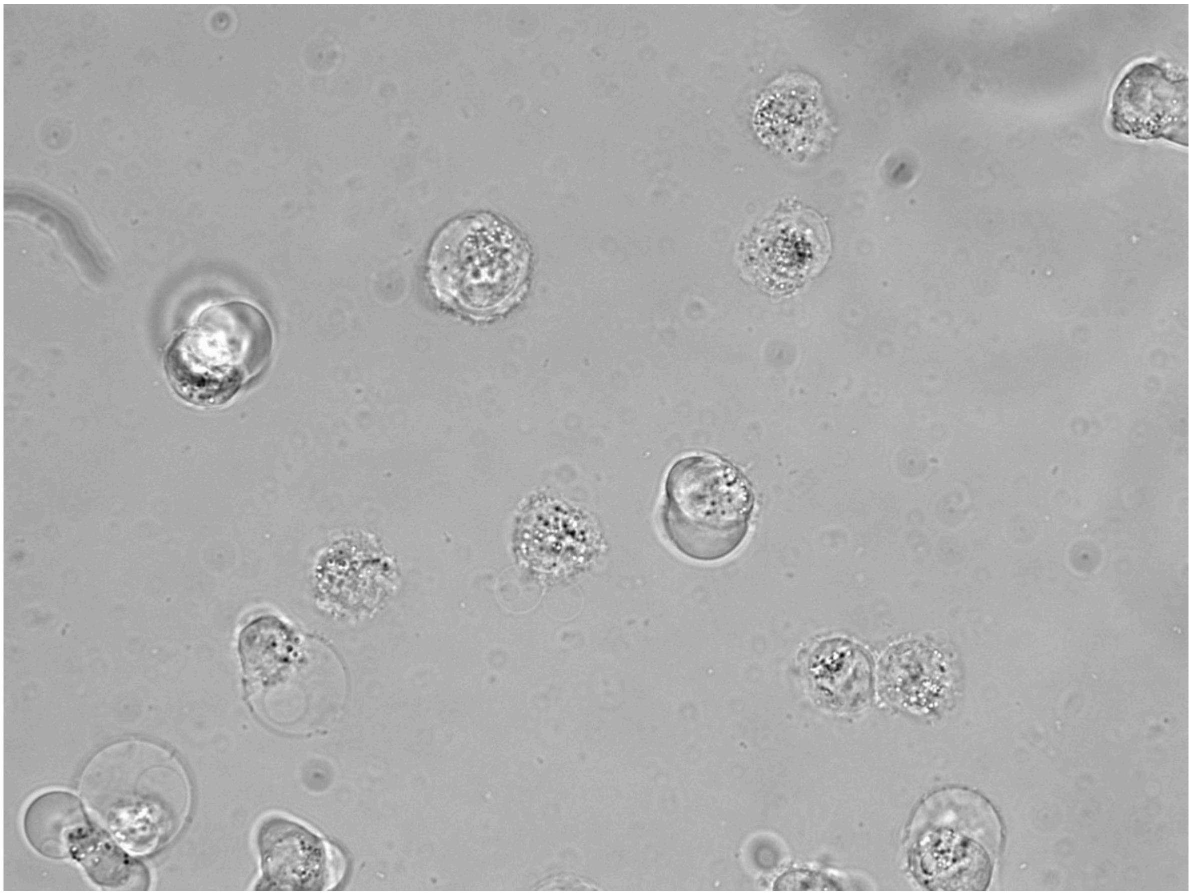

Supplementary Movie 2: Time lapse imaging of MB231 cells treated with ONC201. Cells were treated with DMSO (Ctl.) in RPMI medium supplemented with galactose, and monitored every 20min with for 20h. Note that galactose-containing medium was used instead of glucose-containing medium to capture the ONC201-induced membrane ballooning phenotype in 20 h time frame with live cell imaging system. The same phenotype was observed in 3-5 days in glucose-containing medium (see Figure 1E). Scale bar= 50µm.

See Supplementary File 2

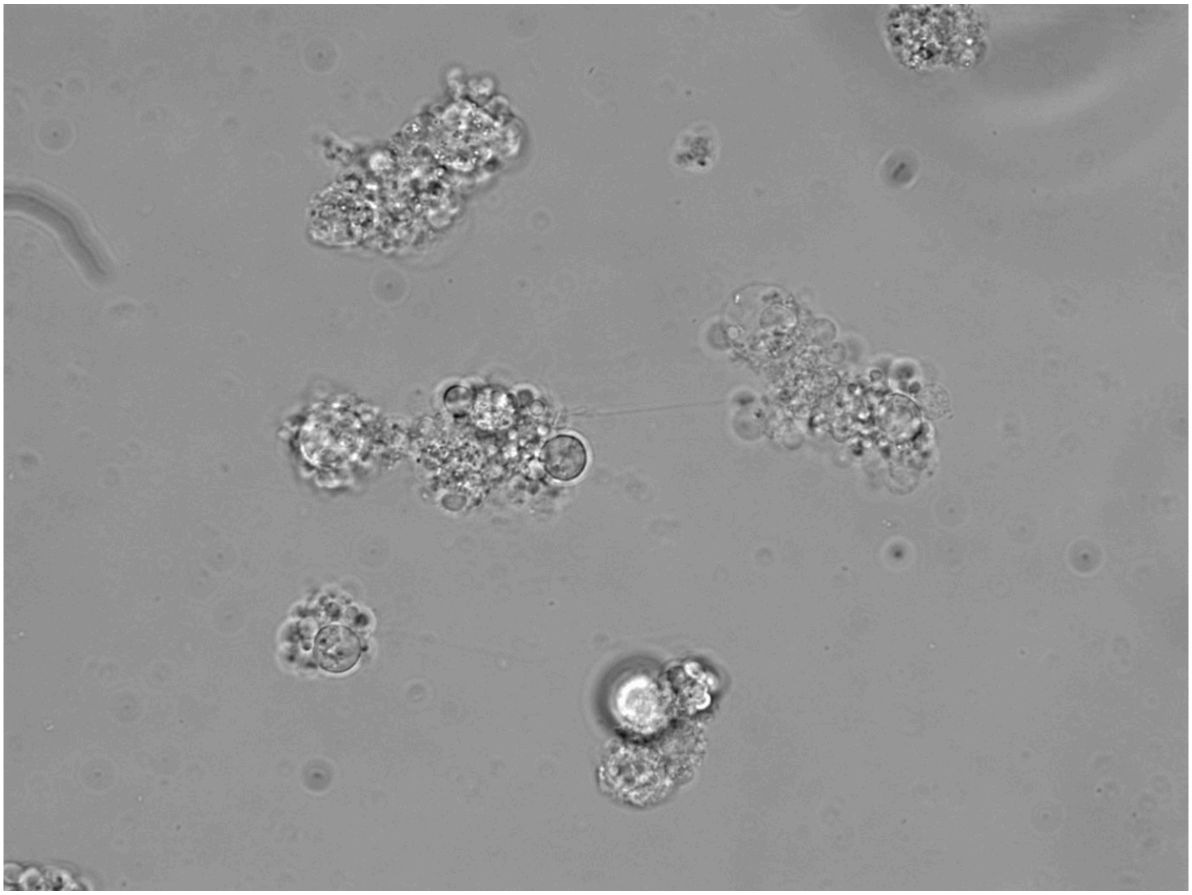

Supplementary Movie 3: Time lapse imaging of MB231 cells treated with GST-TRAIL. Cells were treated with DMSO (Ctl.) in RPMI medium supplemented with glucose, and monitored every 20 min with for 20 h. Scale bar= 50µm.

See Supplementary File 3

Supplementary Table 1: IC50 of ONC201 in cell lines tested

cell type	subtype	cell line	IC50 (uM)#	sensitivity
breast cancer	ER+	T47D	3.29	sensitive
		MCF7	2.4	
		HCC1500	2.55	
		ZR75-1	3.61	
	ER+/HER2+	BT474	1.89	
	HER2+	SKBR3	1.28	
		HCC1954	1.12	
		AU565	0.79	
	TNBC BaA	HCC1937	2.16	
		MDA-MB468	1.24	
		HCC1187	0.78	
		BT20	0.89	
	TNBC BaB	MDA-MB231	2.02	
		Hs578T	0.8	
		MDA-MB436	4.83	
		HCC38	1.91	
		BT549	0.99	
endometrial cancer	serous	ARK1	14.07	
		ARK2	3.046	
	endometrioid	CRL1622	4.285	
		NCI-EC1	2.357	
colorectal carcinoma		HCT116	1.62	
renal cancer	Renal cell carcinoma	UOK121	3.095	
human foreskin fibroblast	N/A	HFF	>20	resistant
renal cancer	hereditary leiomyomatosis renal cell carcinoma	UOK262 FH (-/-)	>20	
rho0 cells	renal cell carcinoma	UOK121* rho0	>20	
	breast cancer	MDA-MB231* rho0	>20	

#IC50 was obtained by at least 3 independent MTS assays in each cell line.

Supplementary Table 2: List of antibodies, siRNA, sequence of PCR primers

Antibody

antibody	cat#	source
Actin	A5316	Sigma-Aldrich
Akt	9272	Cell Signaling
ATF4	11815	Cell Signaling
Caspase3	9662	Cell Signaling
CHOP	2895	Cell Signaling
Cleaved caspase3	9661	Cell Signaling
Cleaved PARP	5625	Cell Signaling
DR4	GTX28414	GeneTex
DR5	sc-65314	Santa Cruz
DR5	2019	ProSci
DRP1	8570	Cell Signaling
EndoG	4969	Cell Signaling
ERK2	sc-154	Santa Cruz
HSC70	sc-7298	Santa Cruz
LC3 A/B	12741	Cell Signaling
Mfn2	9482	Cell Signaling
Mitochondrial oxphos cocktail	MS604-300	Abcam
OPA1	67589	Cell Signaling
PARP	9542	Cell Signaling
phospho-Akt (Ser473)	4058/9271	Cell Signaling
phospho-AMPK alpha (Thr172)	2535	Cell Signaling
Phospho-DRP1 (Ser616)	4494	Cell Signaling
phospho-Foxo3a (Ser253)	9466	Cell Signaling
phospho-Foxo3a (Ser294)	5538	Cell Signaling
Phospho-p44/42 MAPK (Erk1/2) (Thr202/Tyr204)	9101	Cell Signaling
phospho-p70S6K (Thr389)	9234	Cell Signaling
Tfam	8076	Cell Signaling
total-AMPK	5832	Cell Signaling
total-p-70S6K	9202	Cell Signaling

siRNA

target gene	product name	target sequence (5'-3')	cat#	source
DR4	Hs_TNFRSF10A_1	CAGGAACTTTCCGGAATGACA	SI00056728	Qiagen
DR5	Hs_TNFRSF10B_6	CTGCAAATATGGACAGGACTA	SI03094063	Qiagen
Tfam	Hs_TFAM_2	AGGACGAAACTCGTTATCATA	SI00049007	Qiagen
Tfam	Hs_TFAM_4	AAGGAATTATATATTCAGCAT	SI00049021	Qiagen
Tfam	Hs_TFAM_10	CCACGGTGTTCTGTGATATAA	SI04988487	Qiagen
Negative	AllStars Negative		SI03650318	Qiagen
Control	Control siRNA			

PCR primers

target gene	primer sequence	PCR product size
tRNA-Leu (mitochondrial DNA encoded gene)	5' -GATGGCAGAGCCCGGTAATCGC-3'<br>5' -TAAGCATTAGGAATGCCATTGCG-3'	139
POLG (nuclear encoded gene)	5' -AGCGACGGGCAGCGGCGGCGGCA-3'<br>5' -CCCTCCGAGGATAGCACTTGCGGC-3'	92
